# Derivation of mimetic γδ T cells endowed with cancer recognition receptors from reprogrammed γδ T cell

**DOI:** 10.1371/journal.pone.0216815

**Published:** 2019-05-09

**Authors:** Jieming Zeng, Shin Yi Tang, Shu Wang

**Affiliations:** 1 Institute of Bioengineering and Nanotechnology, Singapore; 2 Department of Biological Sciences, National University of Singapore, Singapore; University of Pécs Medical School, HUNGARY

## Abstract

Using induced pluripotent stem cells (iPSCs) to derive chimeric antigen receptor-modified T (CAR-T) cells has great industrial potential. A previous study used αβ T cell-derived CAR-modified iPSCs to produce CAR-T cells. However, these αβ T cells are restricted to autologous use and only recognize single cancer antigen. To make CAR-T alternative for allogeneic use, we reprogrammed γδ T cell into iPSCs (γδ T-iPSCs) to circumvent the risk of graft-versus-host disease. To target multiple cancer-associated antigens, we used an “NK cell-promoting” protocol to differentiate γδ T-iPSCs and to induce expression of natural killer receptors (NKRs). Through such two-step strategy, mimetic γδ T cells endowed with an array of NKRs and thus designated as “γδ natural killer T (γδ NKT) cells” were derived. With no/low-level expression of inhibitory killer cell immunoglobulin-like receptors (KIRs) and immune checkpoint receptors, γδ NKT cells may provide a potent “off-the-shelf” cytotoxic cell source to recognize multiple ubiquitous antigens in a broad spectrum of cancers.

## Introduction

Clinical success of CD19-targeting chimeric antigen receptor-modified T (CAR-T) cells in treating B-cell malignancies symbolizes the translation of synthetic immunology into cellular immunotherapy[1, 2]. To generate unlimited CAR-T cells, Themeli et al. previously described a three-step strategy that combined induced pluripotent stem cell (iPSC) and CAR technologies[3]: Firstly, αβ T cells were reprogrammed to generate αβ T cell-derived iPSCs (αβ T-iPSCs); αβ T-iPSCs were then genetically modified with CAR gene to generate CAR-modified αβ T-iPSCs (CAR-αβ T-iPSCs); lastly, CAR-αβ T-iPSCs were differentiated to generate CAR-T cells. However, such iPSC derivatives express αβ T cell receptors (αβ TCRs) and may cause graft-versus-host disease (GvHD) in allogeneic therapies [4]. To implement such strategy would require generation of a verified CAR-αβ T-iPSC line specifically for every patient and differentiation of this patient-specific iPSC line into customized CAR-T cells, which are expensive, time-consuming and impractical for widespread and timely clinical use. Moreover, while relying on CAR as the sole receptor to recognize single antigen, such CAR-T cells would be impotent when encountering target loss[5] or tumor escape[6]. Here, to address these limitations, we designed a strategy to produce “off-the-shelf” CAR-T alternative from one iPSC line to target multiple antigens in various types of cancer for many patients.

To produce “universal” T-cell therapeutics from iPSCs that serve many recipients with no/low risk of GvHD, a γδ T cell-like end-product is more desirable than a αβ T cell-based one mainly because they do not express αβ TCRs. In humans, 1–10% of peripheral blood lymphocytes are γδ T cells, of which Vγ9Vδ2 T cells are the major subset[7]. Vγ9Vδ2 T cells express Vγ9Vδ2 TCRs to recognize phosphoantigen (PAg) on infected or transformed cells[7]. In contrast to human leukocyte antigen (HLA) -dependent antigen recognition of αβ TCR, PAg recognition of Vγ9Vδ2 TCR is HLA-independent. Hence, Vγ9Vδ2 T cells are unlikely to cause GvHD in an allogeneic setting[8]. Moreover, Vγ9Vδ2 T cells can kill cancer cells even without modification and are being used in clinical trials[9]. To recognize a specific cancer antigen, Vγ9Vδ2 T cells can be transduced to express CAR[10]. Again, just like their αβ T cell-based counterparts, these single antigen-targeting γδ T cells may merely result in “immunoediting” of cancer and eventually cancer escape. To reduce cancer resistance, recognition of multiple targets on cancer cells is highly desirable. Theoretically, it is possible to use multiple CARs to target multiple antigens; however, it is not easy to find an almost ideal surface antigen like CD19 that can be safely targeted without causing serious unmanageable on-target off-cancer side effect, not to mention finding multiple such antigens. Beyond CAR, alternative HLA-independent cancer recognition mechanisms in the form of natural killer cell receptors (NKRs) do exist in the innate immune system[11]. It is well-studied that natural killer (NK) cells possess an array of activating receptors such as NKG2D, DNAM-1, natural cytotoxicity receptors (NCRs) and CD16 to target cancer[11]. Moreover, the cognate ligands of these receptors such as MICA/B and ULBP1-6 are ubiquitously expressed on many types of cancer, but not present on most healthy cells[11]. Safe utilization of this recognition system has been evidenced by the major role of NK cells in immune surveillance against cancer and the clinical trials using NK cells[12]. In Vγ9Vδ2 T cells, NKG2D and DNAM-1 are constitutively expressed[13–16]. However, the expression of NCRs including NKp30, NKp44 and NKp46 were not detected and were not inducible by activation[15, 17–20]. CD16 was only detected in minority of γδ T cells and could not be upregulated by activation[15, 21], while CD56, a marker associated with NK effector function, was detected in about 19% of unstimulated γδ T cells and could only be moderately upregulated by activation[21]. Thus, we envisioned that incorporating such an innate cancer recognition system commonly used by NK cells into iPSC-derived Vγ9Vδ2 T cells would enable them to recognize multiple ubiquitous cancer antigens and enhance their potency to fight against cancers.

## Results

### Designing a synthetic strategy to derive mimetic Vγ9Vδ2 T cells endowed with NKRs from iPSCs

**Fig 1:**
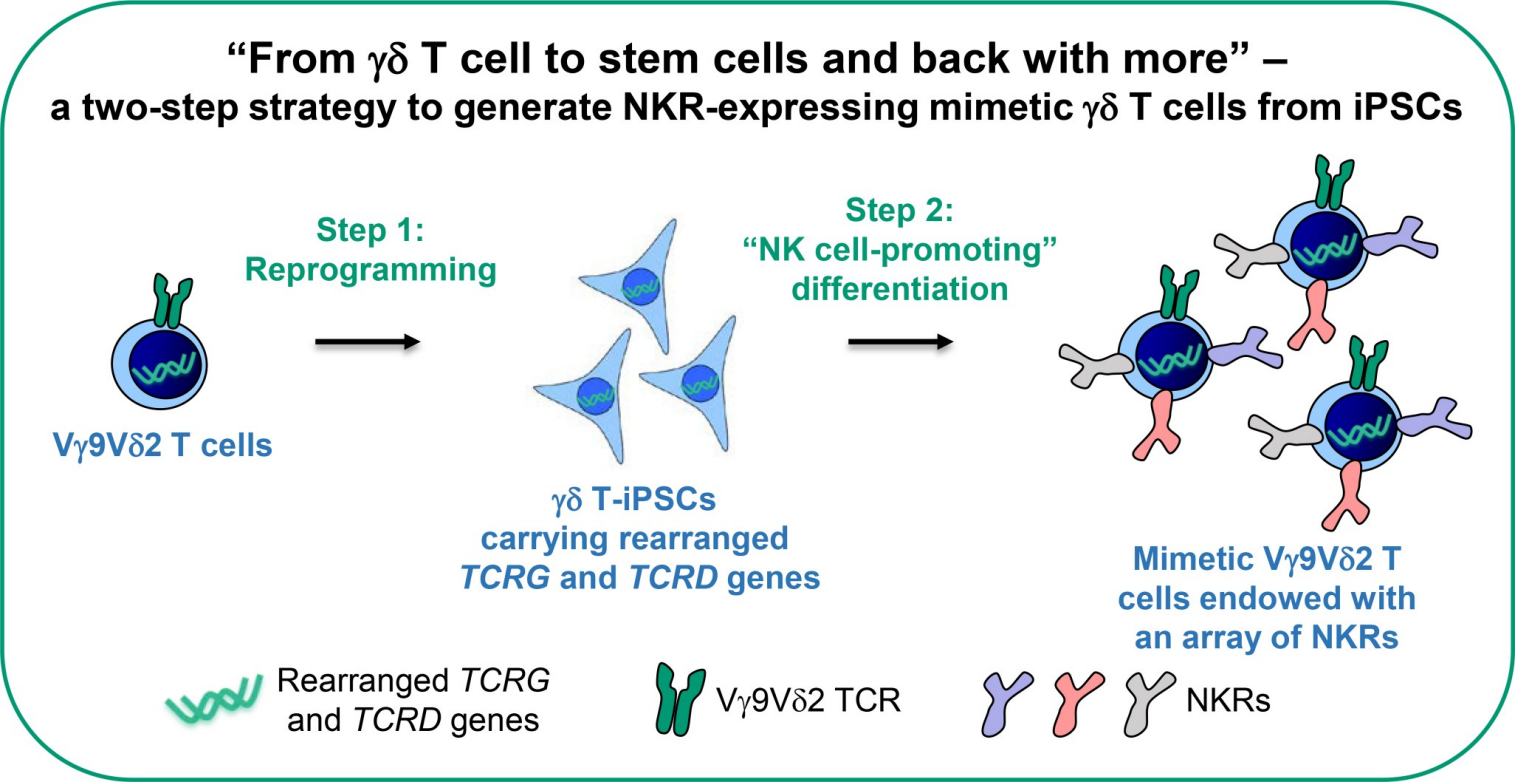
A schematic of a two-step strategy to derive mimetic γδ T cells endowed with NKRs from iPSCs. In step 1, Vγ9Vδ2 T cells are reprogrammed to generate γδ T cell-derived iPSCs (γδ T-iPSCs) carrying the rearranged *TCRG* and *TCRD* genes; in step 2, γδ T-iPSCs are differentiated to Vγ9Vδ2 T cells that express NKRs using an “NK cell-promoting” differentiation protocol.

Rearranged *TCRG* and *TCRD* genes and γδ TCR expression are the hallmarks of γδ T cells[22]. While it is challenging to accurately recapitulate the process of somatic recombination of *TCRG* and *TCRD* genes *in vitro*, previous iPSC technology prompts a possible solution to generate Vγ9Vδ2 T cells from iPSCs. It has been demonstrated that an antigen-specific αβ T cell can be reprogrammed into iPSCs, which will still carry the same rearranged *TCRA* and *TCRB* genes and that such αβ T cell-derived iPSCs can be re-differentiated into αβ T cells, which will re-express the same antigen-specific αβ TCR[23, 24]. Using this strategy, many antigen-specific αβ T cells can be generated from an iPSC line. But the feasibility of using such strategy to generate γδ T cells from γδ T cell-derived iPSCs (γδ T-iPSCs) remains unexplored. Furthermore, to express multiple NKRs in γδ T cells, genetic engineering could be a possible approach. However, limited genetic payload and limited size and number of changes that can be safely made in the genome of an immune cell remain the practical constraints to use such an approach for delivering and integrating multiple genes[2]. We hypothesized that genetic modification might be unnecessary if we were able to induce the expression of NKRs in the process of differentiating γδ T-iPSCs into mimetic γδ T cells. Thus, in view of the above-mentioned possibilities, we designed a simple two-step strategy to generate functionally enhanced mimetic γδ T cells from iPSCs (Fig 1): In step 1, Vγ9Vδ2 T cells are reprogrammed to generate γδ T-iPSCs; in step 2, γδ T-iPSCs are differentiated into NKR-expressing mimetic Vγ9Vδ2 T cells using an “NK cell-promoting” protocol. Here, we demonstrated that this two-step strategy is feasible. The γδ T-iPSC-derived mimetic Vγ9Vδ2 T cells are endowed with an array of NKRs and are potent to target a broad range of cancers.

### Reprogramming Vγ9Vδ2 T cells into γδ T-iPSCs

**Fig 2:**
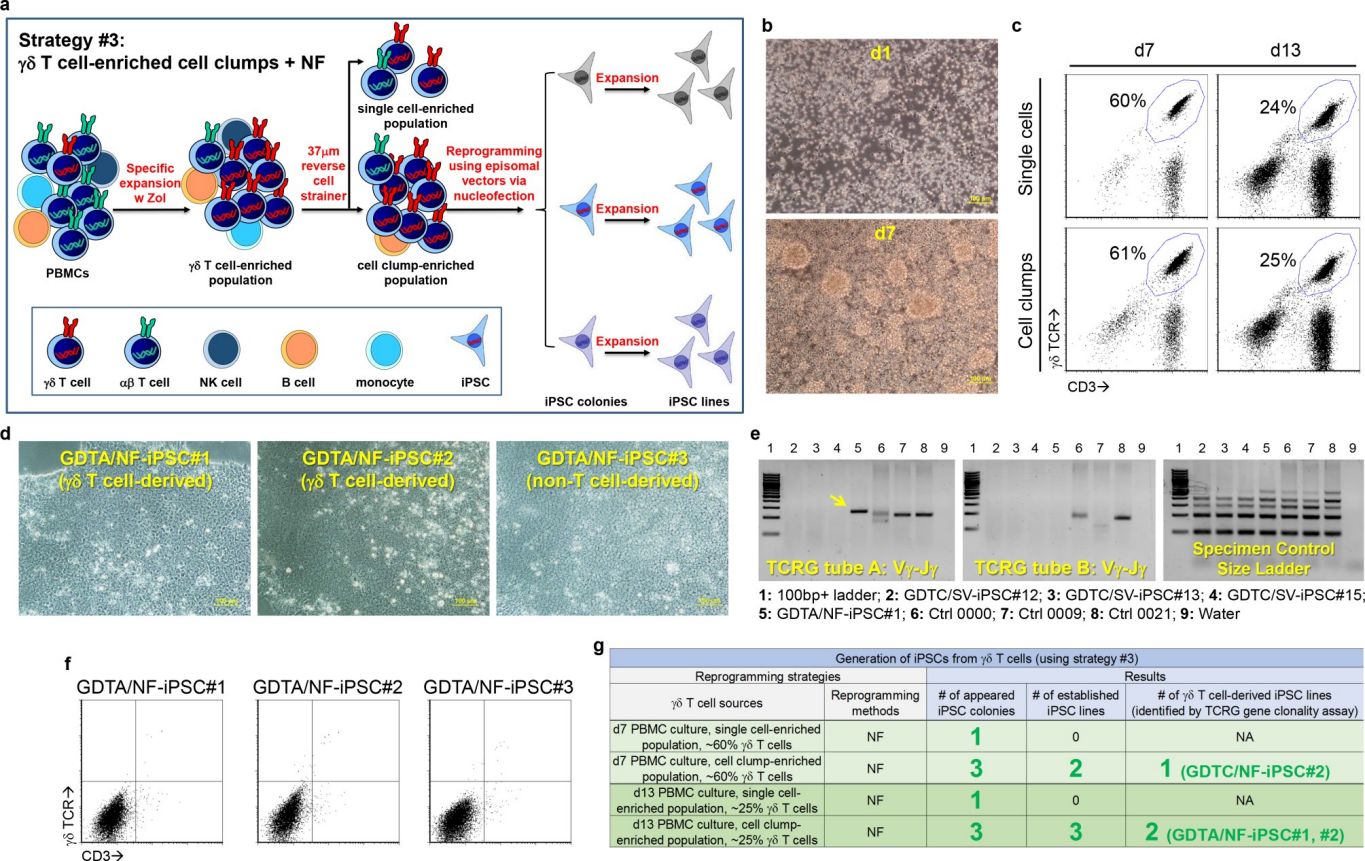
Reprogramming Vγ9Vδ2 T cells into γδ T-iPSCs. (a) A schematic of reprogramming strategy #3 that uses γδ T cell-enriched cell clump population and nucleofection to generate γδ T-iPSCs. (b-c) Morphology (b) and phenotype (c) of PBMCs cultured with zoledronic acid and IL-2. The numbers in the dot plots indicate the % of CD3+γδ TCR+ cells. (d) Morphology of three iPSC lines generated with reprogramming strategy #3. (e) Identification of γδ T-iPSC line using *TCRG* gene clonality assay. The yellow arrow indicates positive amplified product. (f) Detection of CD3 and γδ TCR expression in three iPSC lines by flow cytometry. (g) A result summary of γδ T-iPSC generation using reprogramming strategy #3.

We tested three reprogramming strategies to generate iPSCs from Vγ9Vδ2 T cells (S1 Fig, S2 Fig, Fig 2 and S1 Table). To activate and expand Vγ9Vδ2 T cells for iPSC generation, we cultured peripheral blood mononuclear cells (PBMCs) from a healthy donor using zoledronic acid (Zol) and interleukin-2 (IL-2). Total cell number increased and cell clumps appeared in the PBMC cultures over time (S1B Fig and Fig 2B), which indicate the expanding of Vγ9Vδ2 T cells. More than 60% of one-week cultured cells were γδ T cells, which decreased to less than 30% in two-week cultures (S1C Fig). In strategy #1, high-purity γδ T cells were sorted from the PBMC cultures and transduced with Sendai viral vectors carrying the reprogramming factor genes (S1A Fig). Although these γδ T cells survived cell sorting and Sendai viral transduction (S1B Fig), they did not generate iPSC colonies after seeding onto mouse embryonic fibroblasts (mEFs) (S1D Fig), possibly because of the detrimental effect of high-speed cell sorting on γδ T cells. In strategy #2, to avoid cell sorting and to enhance reprogramming, we used a 37 μm cell strainer to separate cell clump-enriched population and single cell-enriched population because cell clumps may contain more actively proliferating cells and thus facilitate reprogramming (S2 Fig). Indeed, Sendai viral transduction resulted in 17 iPSC colonies and 12 established iPSC lines from cell clump-enriched population, but no colony from single cell-enriched population (S2B–S2D Fig). Surprisingly, *TCRG* gene clonality assay showed that none of these 12 established iPSC lines were derived from γδ T cells (Fig 2E, S2D Fig and S3 Fig), probably due to unknown infection bias of Sendai viral vector. In strategy #3, to avoid the potential viral infection bias, we reprogrammed the cell clump-enriched population using episomal vectors delivered via nucleofection despite its low efficiency (Fig 2). Using this strategy, five iPSC lines were established. Three out of these five iPSC lines were derived from γδ T cells as examined with *TCRG* gene clonality assay (Fig 2E, Fig 2G and S3 Fig). Among these iPSC lines, GDTA/NF-iPSC#1 and GDTA/NF-iPSC#2 were derived from γδ T cells, whereas GDTA/NF-iPSC#3 was from a non-T cell (Fig 2D, Fig 2E and S3 Fig). Like non-T cell-derived GDTA/NF-iPSC#3, γδ T cell-derived GDTA/NF-iPSC#1 and GDTA/NF-iPSC#2 expressed no γδ TCR or CD3 (Fig 2F), suggesting the thorough reprogramming of γδ T cells. PCR and sequencing results showed that GDTA/NF-iPSC#1 contained a *TCRG Vγ9* gene and a *TCRD Vδ2* gene (S4 Fig), confirming its derivation from a Vγ9Vδ2 T cell. Moreover, this iPSC line showed typical human embryonic stem cell-like morphology after expansion (S5A Fig) and expressed pluripotent markers OCT4, SOX2 and NANOG as analyzed by RT-PCR (S5B Fig) as well as surface pluripotent markers SSEA4, TRA-1-60 and TRA-1-8 as detected by immunostaining (S5C Fig). Hereafter, we focused mainly on GDTA/NF-iPSC#1 for most experiments.

### Differentiating γδ T-iPSCs into mimetic Vγ9Vδ2 T cells

**Fig 3:**
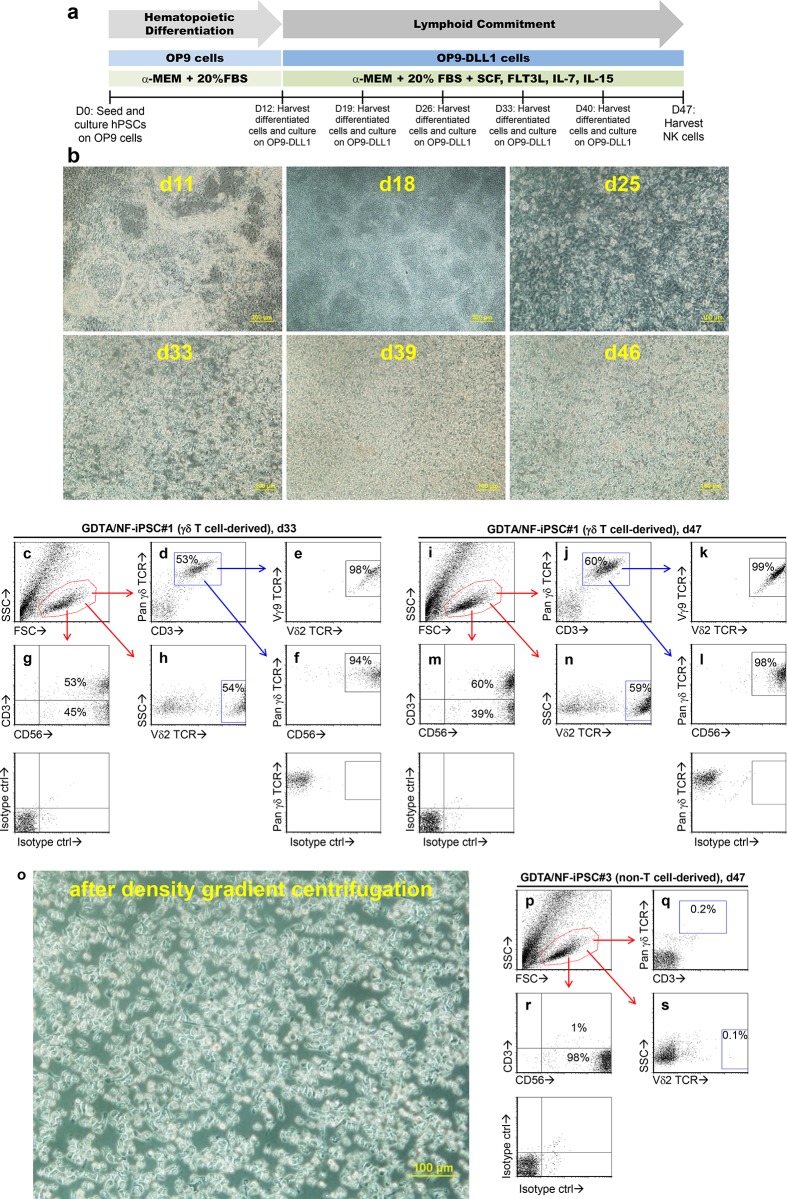
Differentiating γδ T-iPSCs into mimetic Vγ9Vδ2 T cells. (a) A schematic of an established differentiation protocol to generate NK cells from iPSCs. This “NK cell-promoting” protocol was used to generate mimetic Vγ9Vδ2 T cells from γδ T-iPSCs. (b) Morphological changes during differentiation of a γδ T-iPSC line, GDTA/NF-iPSC#1. (c-l) Phenotype of the lymphoid population generated from GDTA/NF-iPSC#1 on d33 and d47 of differentiation as analyzed by flow cytometry. (o) Morphology of a lymphoid population generated from GDTA/NF-iPSC#1 after density gradient centrifugation using Ficoll-Paque. (p-s) Phenotype of the lymphoid population generated from a non-T cell-derived iPSC line, GDTA/NF-iPSC#3 on d47 of differentiation. Isotype controls used for gating CD56+ cells were also included in (f), (g), (l), (m) and r.

To differentiate γδ T-iPSCs into mimetic Vγ9Vδ2 T cells (iPSC-Vγ9Vδ2 T cells), we used our previously established protocol that generates NK cells from iPSCs (Fig 3A)[25]. Fig 3B showed the morphological changes of γδ T-iPSCs during this differentiation process: on d11, GDTA/NF-iPSC#1 attached to and differentiated on OP9 cells after seeding; on d18, ring-like structures were observed after re-seeding onto OP9-DLL1 cells; on d25, bright and round semi-attached cells appeared; on d33, suspension cells appeared and proliferated thereafter. Flow cytometric analysis showed a homogenous lymphoid population in d33 culture (Fig 3C), of which 53% were γδ TCR+CD3+ (Fig 3D). These γδ TCR+CD3+ cells were also Vγ9+Vδ2+ (Fig 3E) and CD56+ (Fig 3F), indicating that they belonged exclusively to the Vγ9Vδ2 T cell subset. This result tallied well with the findings that 53% of the lymphoid population was CD3+CD56+ and 45% was CD3-CD56+ (Fig 3G) and that 54% was Vδ2+ (Fig 3H). In d47 culture, the lymphoid population became more obvious (Fig 3I). More γδ TCR+CD3+ cells appeared (Fig 3J), which remained Vγ9+Vδ2+ (Fig 3K) and CD56+ (Fig 3L). This change was further confirmed by the increase of CD3+CD56+ population and Vδ2+ population and the decrease of CD3-CD56+ population (Fig 3M and 3N). A debris-free lymphoid population could be further obtained after density gradient centrifugation (Fig 3O) and up to 1×10^8^ cells could be generated from 3×10^6^ iPSCs. Using another γδ T-iPSC line, GDTA/NF-iPSC#2, we were also able to derive a lymphoid population that expressed γδ TCRs composed of Vγ9 and Vδ2 chains (S6 Fig). Likewise, when using the non-T cell-derived GDTA/NF-iPSC#3 for differentiation, we did observe an obvious lymphoid population in d47 culture (Fig 3P); however, this population was CD3-CD56+ (Fig 3R) and there were neither γδ TCR+CD3+ cells (Fig 3Q) nor Vδ2+ cells (Fig 3S). These findings strongly suggest that we can generate CD56+ iPSC-Vγ9Vδ2 T cells from a γδ T cell-derived iPSC line, but not from a non-T cell-derived one.

### Phenotyping iPSC-Vγ9Vδ2 T cells

**Fig 4:**
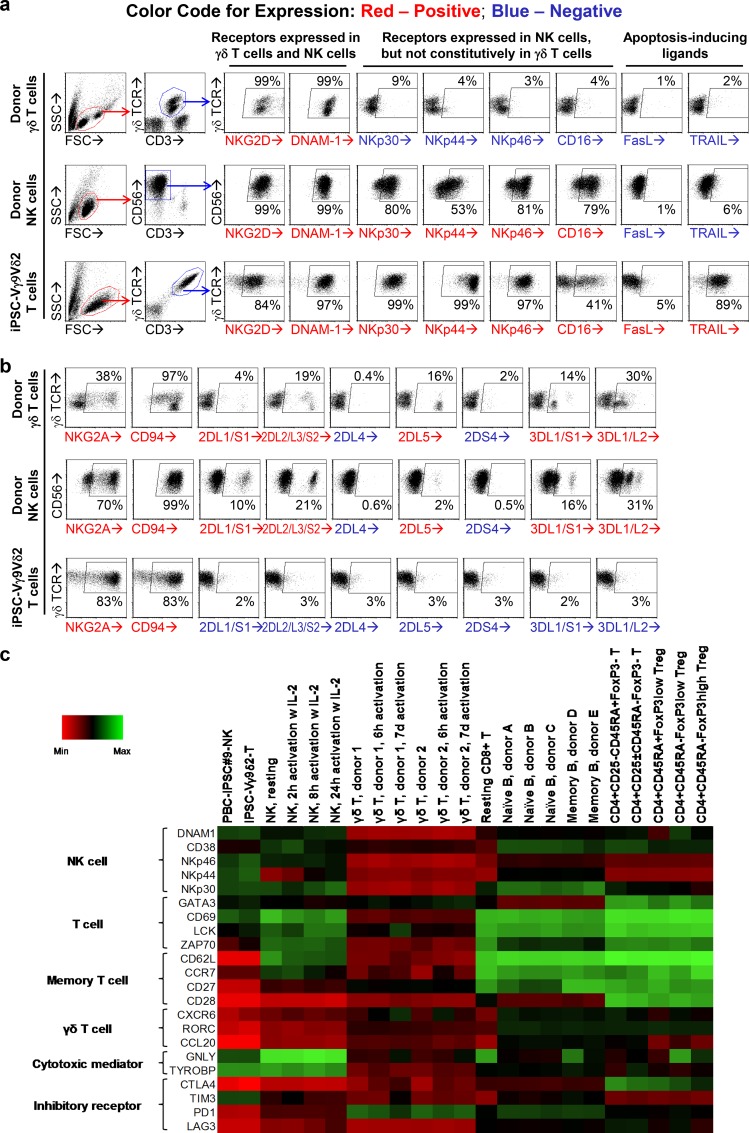
Phenotyping iPSC-Vγ9Vδ2 T cells. iPSC-Vγ9Vδ2 T cells were generated from GDTA/NF-iPSC#1. Donor γδ T cells and donor NK cells were expanded from PBMCs of a healthy donor as controls. Expression of cancer recognition molecules (a) and inhibitory receptor/KIRs (b) on donor γδ T cells, donor NK cells and iPSC-Vγ9Vδ2 T cells were analyzed by flow cytometry. mRNA expression profile of iPSC-Vγ9Vδ2 T cells was analyzed using microarray. A heat map was derived to compare the expression of lymphoid-related genes in iPSC-Vγ9Vδ2 T cells with that in other peripheral blood lymphocytes including γδ T cells, NK cells, CD8+ αβ T cells, B cells, CD4+ αβ T cellsand the previously derived PBC-iPSC-NK cells (c).

CD56 is a typical surface marker of NK cells. CD56 expression on iPSC-Vγ9Vδ2 T cells suggests that these cells may have an NK cell-like phenotype. To find out more, we compared the expression of cancer recognition molecules on iPSC-Vγ9Vδ2 T cells with that on donor γδ T cells expanded as described above for 7 days and donor NK cells expanded as previously described[25](Fig 4A). Flow cytometric analysis showed that donor γδ T cells only expressed a handful of surface receptors: γδ TCR, NKG2D and DNAM-1; while donor NK cells did not express γδ TCR, but expressed NKG2D, DNAM-1, NKp30, NKp44, NKp46 and CD16. In comparison, iPSC-Vγ9Vδ2 T cells expressed all the above-mentioned receptors, which can be categorized into: (1) γδ TCR; (2) activating receptors: NKG2D and DNAM-1; (3) NCRs: NKp30, NKp44 and NKp46; (4) CD16, a surface molecule mediates antibody-dependent cell-mediated cytotoxicity (ADCC); together with (5) apoptosis-inducing ligands: TRAIL and FasL, which are not expressed or weakly expressed in donor γδ T cells and NK cells. In terms of inhibitory receptors and killer cell immunoglobulin-like receptors (KIRs), iPSC-Vγ9Vδ2 T cells expressed CD94/NKG2A receptor just like donor γδ T cells and donor NK cells, which may prevent their overactivation; however, unlike donor NK cells and donor γδ T cells, iPSC-Vγ9Vδ2 T cells did not express KIRs (Fig 4B), which renders them insensitive to inhibition by recipient’s HLA phenotype.

To further compare iPSC-Vγ9Vδ2 T cells with other peripheral blood lymphocytes, e.g. γδ T cells, NK cells, CD8+ αβ T cells, CD4+ αβ T cells and B cells, mRNA expression profiles of these cells were examined with microarray (Fig 4C and S7 Fig). Like donor γδ T cells, iPSC-Vγ9Vδ2 T cells expressed typical T cell-related genes (GATA3, CD69, LCK, ZAP70), but they were distinct from donor γδ T cells in the following aspects: (1) no/low-level expression of γδ T cell-related genes (CXCR6, RORC, CCL20), memory T cell-related genes (CD62L, CCR7, CD27, CD28) and inhibitory receptor genes (CTLA4, PD1, LAG3) and (2) high-level expression of NK cell-related genes (DNAM1, CD38, NKp46, NKp44, NKp30) and cytotoxicity mediator genes (GNLY, TYROBP) (Fig 4C). In terms of mRNA expression profile of the above-mentioned genes, iPSC-Vγ9Vδ2 T cells were closer to donor NK cells and our previously reported PBC-iPSC-NK cells[25] (Fig 4C). A further comparison with donor CD8+ αβ T cells, CD4+ αβ T cells and B cells showed that iPSC-Vγ9Vδ2 T cells were different from non-cytotoxic cells e.g. CD4+ αβ T cells and B cells that have no/low-level expression of cytotoxicity mediators (Fig 4C). Moreover, iPSC-Vγ9Vδ2 T cells expressed no CD4 or CD8β despite some CD8α expression (S8 Fig), suggesting a different phenotype from donor αβ T cells. Hence, iPSC-Vγ9Vδ2 T cells express cancer recognition molecules of both donor γδ T cells and donor NK cells. Armed with γδ TCR and an array of NKRs, these artificial cytotoxic cells are designated as “γδ natural killer T (γδ NKT) cells” and may target a wide variety of cancer cells.

### Direct cytotoxicity, ADCC, recognition specificity and cytotoxic potency of γδ NKT cells against cancer cells

**Fig 5:**
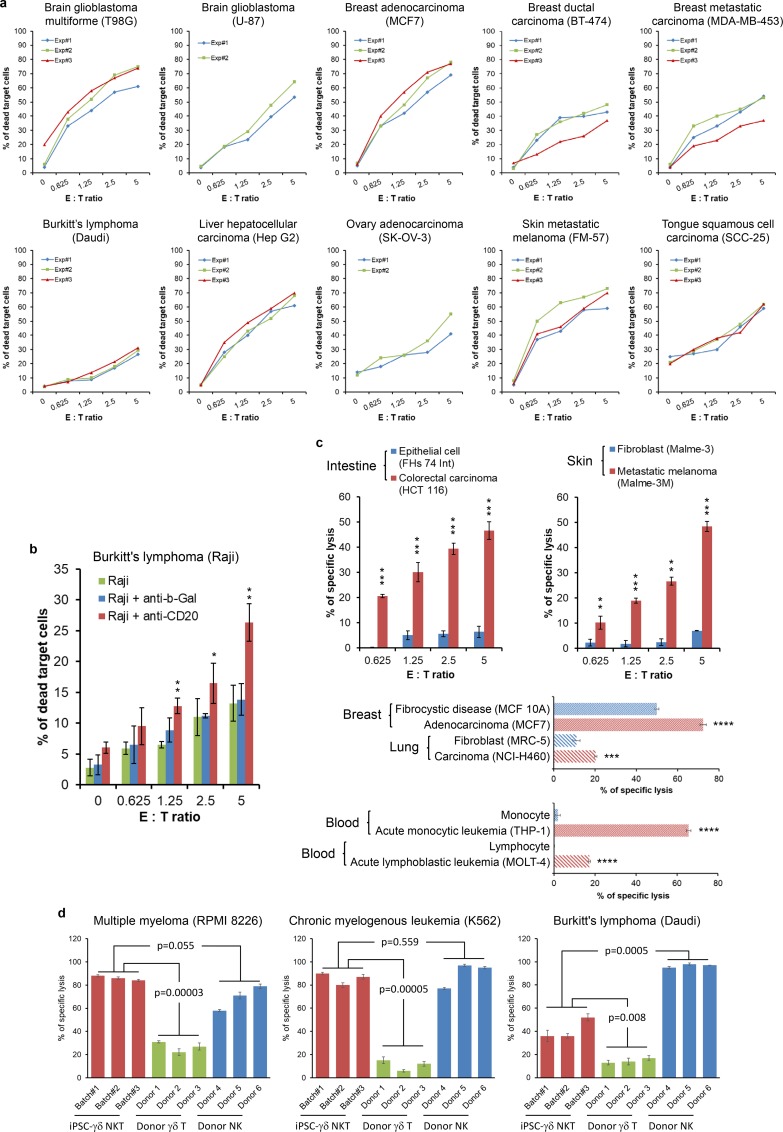
Direct cytotoxicity, ADCC, recognition specificity and cytotoxic potency of γδ NKT cells against cancer cells. (a-b) γδ NKT cells generated from GDTA/NF-iPSC#1 were used for direct cytotoxicity assay against a wide variety of cancer cell lines (a) and ADCC against Raji cells in the presence of humanized anti-CD20 antibody (b). (c) Cancer recognition specificity of γδ NKT cells was investigated by comparing direct cytotoxicity of γδ NKT cells against normal cells or non-tumorigenic cells and their malignant counterparts. The statistical significance of difference in (b) and (c) was determined by Student’s t-test (mean ± SD, n = 3, *p < 0.05, **p < 0.01, ***p < 0.001, ****p < 0.0001). (d) Cytotoxic potency of γδ NKT cells was evaluated by comparing their cytotoxicity against cancer cells with that of donor γδ T cells and donor NK cells. γδ NKT cells from three batches, donor γδ T cells and NK cells from six donors were used in cytotoxicity assay at an E:T ratio of 5. The statistical significance of difference was determined by Student’s t-test (mean ± SD, n = 3).

**Fig 6:**
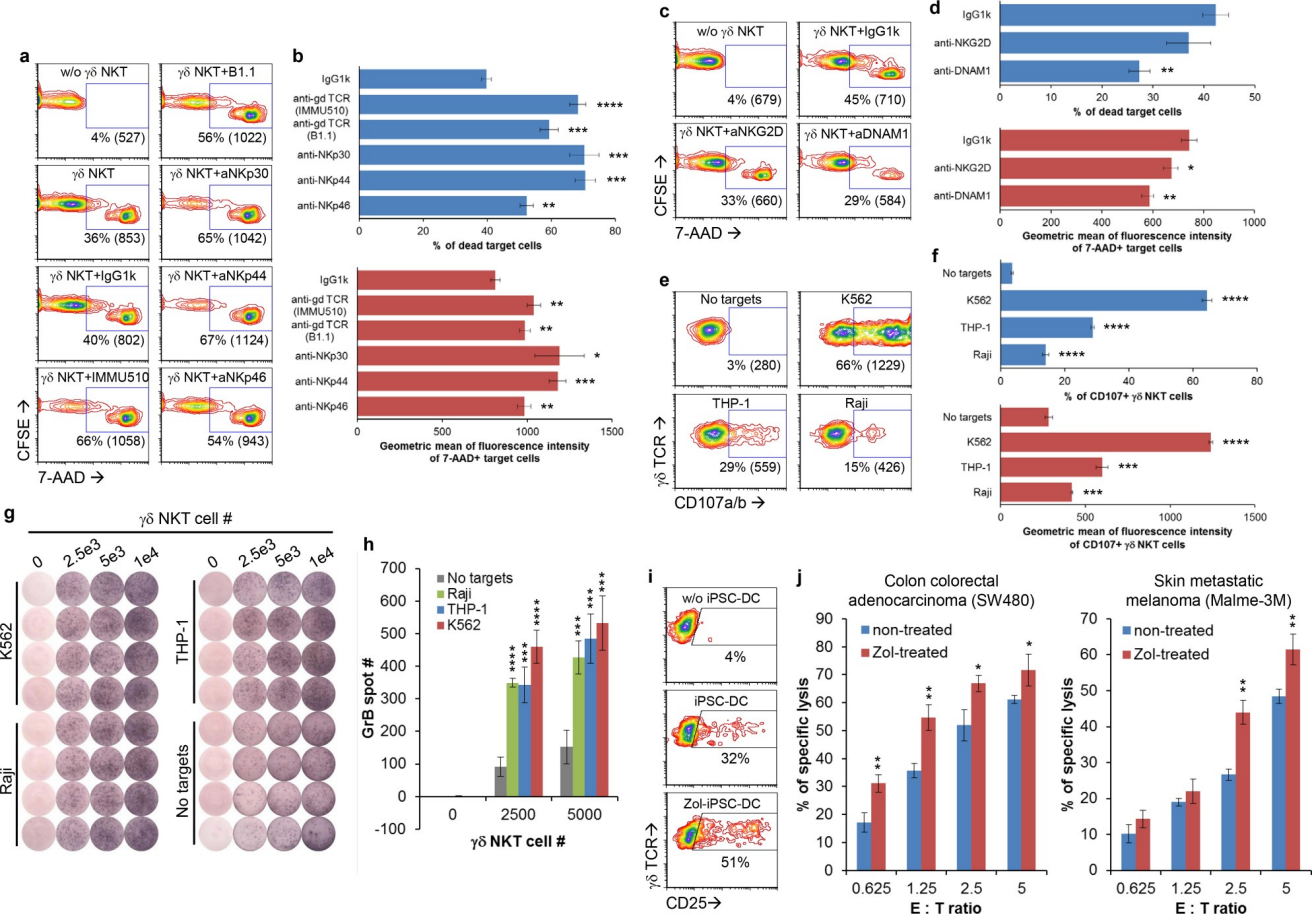
Mechanism of action of γδ NKT cell-mediated cytotoxicity. (a-b) γδ NKT cells generated from GDTA/NF-iPSC#1 were used for redirected cytotoxicity against THP-1 cells in the presence of indicated activating antibodies. (c-d) Cytotoxicity of γδ NKT cells against THP-1 were studied in the presence of indicated blocking antibodies. Representative contour plots (a, c) and result summaries (b, d) were shown. The numbers in contour plots were % of 7-AAD+ THP-1 cells and geometric mean of fluorescence intensity of these cells (numbers in brackets). (e-f) CD107a/b expression on γδ NTK cells after coculture with the indicated cancer cells. Representative contour plots (e) and result summaries (f) were shown. The numbers in contour plots were % of CD107a/b+ γδ NKT cells and geometric mean of fluorescence intensity of these cells (numbers in brackets). (g-h) GrB secretion by γδ NKT cells upon coculture with the indicated cancer cells. ELISPOT images (g) and a summary of spot counting (h) were shown. (i) CD25 expression on γδ NKT cells after stimulation with syngeneic zoledronic acid-treated iPSC-DCs. (j) Cytotoxicity of γδ NKT cells against zoledronic acid-treated cancer cells. The statistical significance of differences while comparing with corresponding controls in above experiments was determined by Student’s t-test (mean ± SD, n = 3, *p < 0.05, **p < 0.01, ***p < 0.001, ****p < 0.0001).

To study whether the unique array of surface receptors on γδ NKT cells can broaden their cancer recognition, we tested direct cytotoxicity of γδ NKT cells against cancer cells of different origins. These cancer cells were as various as follows: glioblastoma of brain (T98G, U-87); adenocarcinoma, ductal carcinoma and metastatic carcinoma of breast (MCF7, BT-474, MDA-MB-453); Burkitt’s lymphoma (Daudi, Raji); hepatocellular carcinoma (Hep G2); adenocarcinoma of ovary (SK-OV-3); metastatic melanoma of skin (FM-57, Malme-3M); squamous cell carcinoma of tongue (SCC-25); colorectal carcinoma and adenocarcinoma of colon (HCT 116, SW480); multiple myeloma (RPMI 8226); chronic myelogenous leukemia (K562); acute monocytic leukemia (THP-1) (Fig 5A, 5C and 5D and Fig 6A–6D and 6J). Results showed that γδ NKT cells efficiently killed these very different cancer cells even at low effector to target (E:T) ratios (Fig 5A and 5C and Fig 6J), suggesting that most surface receptors on γδ NKT cells may be involved in cancer recognition. Direct cytotoxicity of γδ NKT cells against SW480, a colorectal adenocarcinoma cell line was observed under a live imaging microscope. A 48-hour time-lapse video showed that γδ NKT cells eliminated most SW480 cells within first 12 hours (S1 Video), which further confirms the potency of this novel type of killer cells. Another time-lapse video taken in a low cell density setting (S2 Video) clearly showed that the cancer cells were still actively proliferating if there were no attending γδ NKT cells (see the cancer cells in the 4^th^ yellow circle from the left on the top). Death of cancer cells only occurred when there were attending and contacting γδ NKT cells. This has definitively proved that the cytotoxicity of γδ NKT cells against cancer cells depends on cell-cell contact. Moreover, assisted by a humanized anti-CD20 antibody, γδ NKT became more efficient in killing Raji cells (Fig 5B), suggesting CD16 on γδ NKT cells are functional and γδ NKT cells can be used in ADCC.

To study whether γδ NKT cells can still differentiate normal cells from malignant cells while armed with all those receptors, we compared the cytotoxicity of γδ NKT cells against normal cells and their malignant counterparts. Fig 5C showed that γδ NKT cells efficiently killed colorectal carcinoma cells (HCT 116), while sparing normal epithelial cells of intestine (FHs 74 Int). In another example, skin-derived normal fibroblast (Malme-3) and metastatic melanoma (Malme-3M) from a donor were used. γδ NKT cells recognized and killed Malme-3M, but not Malme-3 (Fig 5C). Similarly, γδ NKT cells preferentially killed malignant cells from breast (MCF7), lung (NCI-H460) and blood (THP-1 and MOLT-4) instead of their normal or non-tumorigenic counterparts (MCF 10A, MRC-5, monocyte and lymphocyte, respectively) of the same tissue origin (Fig 5C). These results confirmed the cancer recognition specificity of γδ NKT cells. To understand cytotoxic potency of γδ NKT cells against cancer cells, we compared the direct cytotoxicity of γδ NKT cells with that of donor γδ T cells and donor NK cells against three cancer cell lines (Fig 5D). γδ NKT cells were significantly more potent than donor γδ T cells as demonstrated in all tested lines; the potency was comparable to that of donor NK cells in the tested lines except Daudi (Fig 5D). This finding suggests the importance of NKRs in enhancing cytotoxic potency.

### Mechanism of action of γδ NKT cell-mediated cytotoxicity

To find out whether the surface receptors on γδ NKT cells are involved in cancer recognition and functional in signal transduction, we performed redirected cytotoxicity assay in a monocytic cell line THP-1. Activating antibodies against γδ TCR (both clones IMMU510 and B1.1), NKp30, NKp44 and NKp46 significantly increased cytotoxicity of γδ NKT cells against THP-1 as shown by the increase in % of 7-AAD+ THP-1 cells and geometric mean of fluorescence intensity (MFI) of these cells (Fig 6A and 6B). This result suggests that γδ TCR and all three NCRs are functional in activating γδ NKT cells after crosslinking. To investigate whether NKG2D and DNAM-1 are functional, we used blocking antibodies to interfere signaling through NKG2D and DNAM-1. Blocking DNAM-1 resulted in the decrease of % of 7-AAD+ THP-1 cells and MFI of these cells, while blocking NKG2D led to the decrease in MFI (Fig 6C and 6D), suggesting that both molecules are involved in cancer recognition.

To confirm whether cytotoxicity is mediated through degranulation that releases cytotoxicity mediators, we examined CD107a/b expression on γδ NKT cell surface after 2-hour stimulation with cancer cells. Fig 6E and 6F showed that various cancer cells differentially induced CD107a/b expression on γδ NKT cells with a potency ranking as following: K562 > THP-1 > Raji. ELISPOT assay after 4-hour stimulation further confirmed the release of Granzyme B (GrB) by γδ NKT cells (Fig 6G and 6H). These findings agree with the susceptibility of these cancer cells to γδ NKT cell-mediated cytotoxicity and indicate a major role of degranulation in such process. To study whether Vγ9Vδ2 TCR of γδ NKT cells can recognize PAg, we investigated activation of γδ NKT cells by PAg-presenting cells. To minimize potential allostimulation, we used γδ T-iPSCs to generate syngeneic dendritic cells (iPSC-DCs) as previously described[26–28] to present PAg. Fig 6I showed that Zol-treated iPSC-DCs were more potent than non-treated iPSC-DCs in inducing CD25 expression in γδ NKT cells. Moreover, Zol-treatment also increased the susceptibility of cancer cells (SW480 and Malme-3M) to γδ NKT cell-mediated cytotoxicity (Fig 6J), but not the susceptibility to iPSC-derived NK cells (S9 Fig), which further supports PAg recognition by γδ NKT cells.

## Discussion

Despite current dominance of using autologous αβ T cells to generate CAR-T cells, using iPSCs to generate CAR-T cells may have greater industrial potential due to the possibility to produce homogenous cellular therapeutics in large-scale. Previously, Sadelain’s group demonstrated the use of αβ T cell-derived CAR-modified iPSCs to generate CAR-T cells via a three-step strategy[3]. However, expression of αβ TCRs in such produced CAR-T cells excludes their allogeneic use. Recently, to produce “off-the-shelf” CAR-T cells from αβ T cell-derived iPSC line, the same group used CRISPR/Cas9 to insert CAR gene into the *T-cell receptor* α *constant (TRAC)* locus[29] to eliminate αβ TCR expression. Such engineered iPSC line enables the production of CAR-targeted, TCR-null CD8αβ+ T cells for “off-the-shelf” administration.

Here, to avoid the hassle of genetic manipulation, we demonstrated a distinct two-step approach to generate “off-the-shelf” CAR-T alternative from iPSCs. Firstly, to avoid GvHD from the very beginning and to produce iPSC-derived cytotoxic cells for allogeneic use, we generated iPSC line from γδ T cell instead of αβ T cell for two key advantages: (1) γδ T-iPSC line obviates the need for *TRAC* gene disruption, which is essential for αβ T-iPSC line; (2) γδ T-iPSC line carries rearranged *TCRG* and *TCRD* genes to express γδ TCRs (Vγ9Vδ2 TCRs) for PAg recognition. We have successfully reprogrammed γδ T cells into iPSCs using episomal vectors delivered via nucleofection, while Watanabe et al. have reported recently the use of Sendai virus at high MOI for reprogramming[30]. However, no generation of cytotoxic cells from their γδ T cell-derived iPSCs was carried out in their study[30]. Secondly, to introduce multiple HLA-independent non-CAR cancer recognition receptors without genetic manipulation, we used an “NK cell-promoting” protocol to differentiate γδ T-iPSCs. The resulting “γδ NKT cells” express not only Vγ9Vδ2 TCRs but also five other well-known NKRs (NKp30, NKp44, NKp46, NKG2D and DNAM1), all of which are involved in cancer recognition. γδ NKT cells also come with CD16 to mediate ADCC and death receptor ligands (FasL and TRAIL) to induce apoptosis in cancer cells. Functionally, γδ NKT cells can efficiently recognize and kill a wide variety of cancer cells via degranulation of cytotoxicity mediators. Hence, deriving from γδ T-iPSCs, γδ NKT cells may provide an unlimited “off-the-shelf” cytotoxic cell source with a plethora of built-in cancer recognition mechanisms.

One bottleneck to expand CAR-T therapy beyond B-cell malignancies is the lack of surface antigens that can be safely targeted in cancers especially in solid tumors[1, 2]. In blood cancers, some potential targets other than CD19 are being tested in clinical trials, such as targeting BCMA in multiple myeloma[31]. But in solid tumors, finding specific surface targets is more challenging since most tumor antigens are intracellular and thus not targetable by CAR. Moreover, to overcome cancer recurrence due to antigen loss, simultaneously targeting multiple antigens is critical. However, such a feature remains to be developed for CAR-T platform due to the lack of suitable targets and the limited genetic payload[1, 2]. To target multiple antigens, we exploited the cancer sensing mechanisms of innate immune cell populations. Specifically, Vγ9Vδ2 TCR recognizes upregulated PAg in stressed and transformed cells[7]. NKG2D recognizes NKG2D ligands such as ULBP1-6 and MICA/B that are upregulated in many types of cancer cells[32]. NKp30 recognizes BAT3/BAG6 and B7-H6; NKp44 recognizes NKp44 ligand, a unique splice variant isoform of MLL5 protein; NKp46 recognizes vimentin; and each of these NCRs also recognizes particular heparan sulfate glycosaminoglycans that are uniquely expressed in tumor microenvironments[33]. DNAM-1 recognizes PVR (CD155) and Nectin-2 (CD112) and such interactions are essential for NK cell-mediated cytotoxicity[32]. More importantly, these ligands are induced by dysregulated mevalonate pathway, dysregulated proliferation and/or DNA-damage response and thus preferentially/exclusively expressed on cancer cells[34, 35]. Unlike the lineage-specific antigens (e.g. CD19 of B-cell lineage) targeted by current CAR-T technology, the above-described stress-induced signals represent a restricted set of conserved endogenous surface molecules that are commonly observed in many types of cancer cells and can be safely targeted as evidenced by the innate immune response to cancers. Simultaneously targeting these ubiquitous ligands may not only reduce the risk of cancer escape but also broaden the spectrum of cancer recognition. In this study, we demonstrated that γδ NKT cells generated from γδ T-iPSCs express multiple receptors of innate immune cells to fulfill such cancer recognition requirement.

In current CAR-T platform, the backbone cells are predominately αβ T cells, which are still many steps away from being competent anti-cancer cytotoxic cells[1, 2]. Genetically “hacking” αβ T cells using lentiviral vectors along with gene editing technologies is a possible approach to insert desirable exogenous genes, e.g. multiple CAR genes and to disrupt undesirable endogenous genes, e.g. *TRAC* gene (to avoid GvHD) and *PD-1* gene (to resist inhibition by PD-L1)[36, 37]. However, to make all these events of genetic manipulation happen simultaneously and efficiently in the highly differentiated αβ T cells is technically challenging. Such intensively manipulated immune cells may become immunogenic and induce host immunity that affects their *in vivo* survival[38]. The effects of potential off-target gene insertion and disruption may also be long-term safety concerns. To avert over-reliance on genetic manipulation, it is sensible to begin with backbone cytotoxic cells that come with the above-mentioned features. Such cells may not naturally exist in humans, but they may be generated from iPSCs via a two-step strategy as demonstrated in this study. Through a simple combination of a unique iPSC source γδ T-iPSC and an “NK cell-promoting” differentiation protocol, we can generate cytotoxic γδ NKT cells which are endowed with those desirable features without genetic modification. More specifically, the key features of γδ NKT cells include: (1) they are born with cancer recognition mechanisms of both γδ T cells and NK cells; (2) they are derived from γδ T-iPSC and render no risk of GvHD in allogeneic applications; (3) they have no/low-level expression of inhibitory KIRs and are unrestricted by recipient’s HLA phenotype; (4) they have no/low-level expression of immune checkpoint receptors (e.g. PD-1, CTLA-4 and Lag-3) and are insensitive to immune regulation by cancer cells. Thus, besides “hacking” existing immune cells, deriving from iPSCs may provide alternative strategy to generate potent anti-cancer cytotoxic cells. Combining with other immune intervention strategies such as preconditioning cancer patients with lymphodeleting chemotherapy regimens, which has been commonly used in CAR-T therapies[38–40], may further prolong the *in vivo* survival of such allogeneic cells and thus enhance their clinical potential. Apparently, exactly like any other similar technologies at their early stage, further development of a clinically compliant manufacturing protocol is a prerequisite to fulfill such cancer-fighting potential in clinical applications.

## Methods

### Cell culture

All iPSC lines were cultured with mTeSR1 (StemCell Technologies, Vancouver, BC, Canada, http://www.stemcell.com) on Matrigel (BD Biosciences, San Diego, CA, http://www.bdbiosciences.com) -coated 6-well plates. Cell lines OP9, T98G, U-87, MCF7, BT-474, MDA-MB-453, Daudi, Hep G2, SK-OV-3, SCC-25, Raji, FHs 74 Int, HCT 116, Malme-3, Malme-3M, RPMI 8226, K562, THP-1, SW480, MCF 10A, MRC-5, NCI-H460 [American Type Culture Collection (ATTC), Manassas, VA, http://www.atcc.org] were cultured as recommended by ATCC. Cell line FM-57 [European Collection of Authenticated Cell Cultures (ECACC)] was culture cultured in RPMI 1640 with 10% fetal bovine serum (FBS, Thermo Fisher Scientific, Waltham, MA, http://www.thermofisher.com). Cell line OP9-DLL1 (Riken BRC Cell Bank, Ibaraki, Japan, http://cell.brc.riken.jp/en/) was cultured in MEMα (Thermo Fisher Scientific) with 20% FBS. Frozen human peripheral blood CD14+ monocytes (Lonza, http://www.lonza.com) were thawed and maintained in RPMI 1640 with 10% FBS.

### Generation of γδ T-iPSCs

To expand γδ T cells for iPSC generation, frozen PBMCs from a healthy donor (StemCell Technologies) were thawed and cultured in PBMC culture medium [CTS OpTmizer T-Cell Expansion SFM (Thermo Fisher Scientific) with 10% heat-inactivated human AB serum (Gemini Bio-Products, West Sacramento, CA, http://www.gembio.com) and 10 ng/ml IL-2 (Thermo Fisher Scientific)] containing 5 μM zoledronic acid (Sigma-Aldrich, St Louis, MO, http://www.sigmaaldrich.com). Half medium was replaced with fresh PBMC culture medium without zoledronic acid every 2–3 days and the cultured PBMCs were used for reprogramming after 1 to 2 weeks.

In reprogramming strategy #1, γδ T cells in the PBMC cultures were sorted using a FACS Aria flow cytometer (BD Biosciences) and transduced with Sendai reprogramming vectors from a CytoTune iPS 2.0 Sendai Reprogramming Kit (Thermo Fisher Scientific) at MOI of 5:5:3 (KOS, hc-Myc, hKlf4) overnight. The transduced cells were then washed and cultured for 5 days before seeding to a 6-well plate grown with mitomycin C (Sigma-Aldrich) -treated mEFs. Half medium was replaced on day 1 to 3 after seeding with iPSC medium [DMEM/F12 (Thermo Fisher Scientific) with 20% knockout serum replacement (Thermo Fisher Scientific), 2 mM L-glutamine, 1% nonessential amino acids, 0.1 mM 2-mercaptoethanol and 5 ng/ml basic fibroblast growth factor (PeproTech)]. In strategy #2, the PBMC culture was separated into cell clump-enriched population and single cell-enriched population using a 37 μm reverse cell strainer (StemCell Technologies) for reprogramming with Sendai viral vectors as described above. In strategy #3, these two cell populations were reprogrammed using a nonviral method as following: On day 0, episomal reprogramming vectors from a Epi5 Episomal iPSC Reprogramming Kit (Thermo Fisher Scientific) were delivered into the cells via nucleofection using a Amaxa Nucleofector 2b (Lonza). The nuleofected cells were then seeded on mitomycin C-inactivated mEFs. On day 2, the cells were adapted to a 1:1 mixture of PBMC culture medium: iPSC medium. From day 3 on, the cells were cultured in iPSC medium, which was changed every other day. Two to four weeks after seeding, iPSC colonies were picked up and expanded in Matrigel-coated plates in mTeSR1.

### TCRG gene clonality assay and PCR

To identify γδ T cell-derived iPSC lines, genomic DNA was isolated using a DNeasy Blood and Tissue Kit (Qiagen, Hilden, Germany, https://www.qiagen.com) according to the manufacturer’s instruction. To detect *TCRG* gene rearrangement in genomic DNA, PCR was carried out with master mixes provided in TCRG Gene Clonality Assay kit (Invivoscribe Technologies, San Diego, CA, http://www.invivoscribe.com) and AmpliTaq Gold DNA polymerase (Thermo Fisher Scientific) using the following program: 95ºC for 7 minutes; 35 amplification cycles (95ºC for 45 seconds, 60ºC for 45 seconds, 72ºC for 90 seconds); and final extension of 72ºC for 10 minutes before holding at 15ºC. PCR products were separated by electrophoresis in 2% MetaPhor Agarose (Lonza) gel.

To further confirm the origin of γδ T-iPSC line, genomic DNA was used as PCR template. Primer pairs (TRGV9for: 5'-GCA GGT CAC CTA GAG CAA CC -3' and TRGJPrev: 5'-TGT AAT GAT AAG CTT TGT TC-3') and (TCRVδ2_Fwd: 5'-ATACCGAGAAAAGGACATCTATG-3' and TCRJδ1_Rev: 5'-GTTCCACAGTCACACGGGTTC-3') were used to amplify rearranged *TCRG* and *TCRD* respectively. The amplicons were analyzed on 1% agarose gel and sequenced afterward.

### Differentiation of γδ T-iPSCs into mimetic Vγ9Vδ2 cells

To generate mimetic Vγ9Vδ2 T cells from γδ T-iPSCs, we used a two-stage protocol previously established for generating NK cells from iPSCs[25]. In the first stage, 1–1.5×10^6^ γδ T-iPSCs were seeded and differentiated on overgrown OP9 cells in MEMα supplemented with 20% FBS for 12 days. The cocultures were fed every 4 days by changing half medium. In the second stage, the cocultured cells were harvested using 1 mg/ml collagenase IV (StemCell Technologies) and TrypLE Express (Thermo Fisher Scientific). OP9 cells were removed by plastic adherence for 45 minutes and the cell clumps were further removed by a 100 μm cell strainer (BD Biosciences). The remaining non-adherent cells were then cocultured with OP9-DLL1 cells grown on T75 flasks using MEMα containing 20% FBS, 10 ng/ml SCF (PeproTech), 5 ng/ml FLT3L (PeproTech), 5 ng/ml IL-7 (PeproTech) and 10 ng/ml IL-15 (PeproTech) for 7 days. Hereafter, the differentiated cells were harvested using Versene (Thermo Fisher Scientific) and cocultured with new OP9-DLL1 cells grown on 6-well plates on a weekly basis for another 4 weeks.

### Flow cytometry

To study phenotypic change during γδ T-iPSC differentiation, differentiated cells were harvested and stained using antibodies against CD3, γδ TCR, Vδ2 TCR, Vγ9 TCR, CD56, NKG2D, DNAM-1, NKp30, NKp44, NKp46, CD16, FasL, TRAIL, NKG2A, CD94, CD158a,h (KIR2DL1/S1), CD158b (KIR2DL2/L3/S2), CD158d (KIR2DL4), CD158f (KIR2DL5), CD158i (KIR2DS4), CD158e1/e2 (KIR3DL1/S1) and CD158e/k (KIR3DL1/L2) (BD Biosciences; Beckman Coulter, https://www.beckmancoulter.com; Miltenyi Biotec, http://www.miltenyibiotec.com) and analyzed with a FACS Calibur flow cytometer (BD Biosciences).

### Microarray

To compare the gene expression of γδ NKT cells with other peripheral blood lymphocytes, total RNA of sorted γδ NKT cells was extracted using TRIzol reagent (Thermo Fisher Scientific). RNA quality was assessed with a 2100 Bioanalyzer (Agilent Technologies, Palo Alto, CA, https://www.agilent.com). 150ng of total RNA was used to generate complementary RNA which was then biotinylated and fragmented. The fragmented labeled complementary RNA was then hybridized with GeneChip Human Genome U133 Plus 2.0 Array (Thermo Fisher Scientific). The array was washed, stained and scanned using a GeneChip Fluidics Station 450 and a GeneChip Scanner 3000 7G (Thermo Fisher Scientific). All procedures were performed according to the manufacturer’s standard protocol in the microarray facility at Institute of Molecular and Cell Biology, A*STAR, Singapore. The resulting raw data were compared with the raw data of other peripheral blood lymphocytes downloaded from Gene Expression Omnibus (GEO) database of National Center for Biotechnology Information (NCBI), which include γδ T cells (GSE27291), NK cells (GSE8059), CD8+ αβ T cells (GSE8059), B cells (GSE12195) and CD4+ αβ T cells (GSE15659). To allow comparison across arrays, Expression values were normalized with robust multi-array average (RMA) using an Affymetrix Expression Console Software (Thermo Fisher Scientific). The percentage of gene analysis of variance (ANOVA) expressed on array was calculated using the number of probe sets labelled present or marginal based on an applied algorithm. Subsequently, the comparison between different cells on selected genes was performed using an Affymetrix Transcriptome Analysis Console Software (Thermo Fisher Scientific) for cluster analysis and relative gene expression.

### Direct cytotoxicity, ADCC, redirected cytotoxicity and antibody-blocking assay

A flow cytometry-based method was used to detect direct cytotoxicity of γδ NKT cells against cancer cells. In brief, 0 to 10^5^ γδ NKT cells were cocultured with 2×10^4^ carboxyfluorescein diacetate succinimidyl ester (CFSE; Thermo Fisher Scientific) -labelled target cells at the indicated effector to target (E:T) ratios for 4–6 hours. Samples were then stained on ice with 7-Amino-Actinomycin D (7-AAD, BD Biosciences) for 10 minutes. After washing, target cell death was assessed with flow cytometer by the percentage of 7-AAD-stained cells in CFSE-positive population. In some experiments, target cells were treated with 5μM zoledronic acid overnight prior to cytotoxicity assay. To evaluate ADCC of γδ NKT cells, cocultures of γδ NKT cells and CFSE-labelled Raji cells were set up at the indicated E:T ratios in the presence of 1 μg/ml anti-β-hIgG1 (InvivoGen, San Diego, CA, http://www.invivogen.com) or anti-CD20-hIgG1 (InvivoGen). Raji cell death was measured after 4-hour incubation by flow cytometry as described above.

To study the function of surface receptors on γδ NKT cells, redirected cytotoxicity of γδ NKT cells against CFSE-labelled THP-1 was performed at an E:T ratio of 1 in the presence of 2 μg/ml of mouse IgG1κ (eBioscience, http://thermofisher.com/ebioscience), anti-γδ TCR (IMMU510, Beckman Coulter, Brea, CA, http://www.beckmancoulter.com), anti-γδ TCR (B1.1, eBioscience), anti-NKp30 (BioLegend, San Diego, CA, https://www.biolegend.com), anti-NKp44 (BioLegend) and anti-NKp46 (BioLegned). To investigate the function of NKG2D and DNAM-1 on γδ NKT cells, γδ NKT cells and CFSE-labelled THP-1 were cocultured at an E:T ratio of 1 in the presence of 10 μg/ml blocking antibodies against NKG2D (eBioscience) and DNAM-1(abcam, Cambridge, UK, http://www.abcam.com). THP-1 cell death was measured after 4-hour incubation by flow cytometry.

### Degranulation assay and ELISPOT assay

To study the degranulation of γδ NKT cells, γδ NKT cells were coculture with various target cells at an E:T ratio of 1 in the presence of anti-CD107a (eBioscience), anti-CD107b (eBioscience) and 2 μM monensin (eBioscience). After 2-hour incubation, samples were stained with anti-γδ TCR (Beckman Coulter) and analyzed with flow cytometer.

To measure GrB secretion, a Human Granzyme B ELISpot Kit (R&D Systems, Minneapolis, MN, https://www.rndsystems.com) was used. In brief, 0 to 10^4^ γδ NKT cells were incubated with 5×10^4^ various cancer cells on a human GrB microplate for 4 hours. GrB spots were then stained as described in the manufacturer’s manual and counted with an ImmunoSpot Analyzer (CTL, Shaker Heights, OH, http://www.immunospot.com).

## Supporting information

S1 Fig Strategy #1 to reprogram Vγ9Vδ2 T cells into iPSCs. (a) A schematic of reprogramming strategy #1 that uses sorted high-purity γδ T cells and Sendai viral vectors to generate γδ T-iPSCs. (b-c) Morphology (b) and phenotype (c) of PBMCs cultured with zoledronic acid, post-sort γδ T cells and γδ T cells after Sendai viral transduction. (d) A result summary of iPSC generation using reprogramming strategy #1.
(TIF)

S2 Fig Strategy #2 to reprogram Vγ9Vδ2 T cells into iPSCs. (a) A schematic of reprogramming strategy #2 that uses γδ T cell-enriched cell clump population and Sendai viral vectors to generate γδ T-iPSCs. (b) Phenotype of single cell-enriched population and their morphology after Sendai viral transduction. (c) Phenotype of cell clump-enriched population, their morphology after Sendai viral transduction and the resulting iPSC colony. (d) A result summary of iPSC generation using reprogramming strategy #2.
(TIF)

S3 Fig Identification of γδ T-iPSC lines using TCRG gene clonality assay. To identify iPSC lines derived from γδ T cells, genomic DNA was extracted and PCR was carried out using the master mixes provided in the TCRG gene clonality assay kit. The yellow arrows indicate positive amplified products.
(TIF)

S4 Fig Verification of γδ T-iPSC origin. To confirm the origin of γδ T-iPSC line, genomic DNA was extracted as template. PCR was carried out using primers specific for rearranged TCRG (a) and TCRD (b). The sequences of amplicons were compared with the ones in Gene database at NCBI.
(TIF)

S5 Fig Characterization of γδ T-iPSCs. (a) A high resolution image of a γδ T-iPSC line, GDTA/NF-iPSC#1. (b) Expression of pluripotent markers OCT4, SOX2 and NANOG in GDTA/NF-iPSC#1 as analyzed by RT-PCR. Fibroblast-like cells (FLCs) derived from iPSC lines, GDTA/NF-iPSC#1 and PBC-iPSC#9, using a previously reported protocol (*J Biosci Bioeng*, *120*: *210*, *2015*) were used as controls. (c) Expression of pluripotent markers as detected by immunostaining.
(TIF)

S6 Fig Differentiating γδ T-iPSCs into mimetic Vγ9Vδ2 T cells. Phenotype of the lymphoid population generated from GDTA/NF-iPSC#2 on d47 of differentiation as analysed by flow cytometry.
(TIF)

S7 Fig mRNA expression profile of iPSC-Vγ9Vδ2 T cells analyzed with microarray. (a) Normalized data of microarray for all genes and cell types shown in the heat map in Fig 4C. (b) Coefficient of correlation between the samples. p-values are shown in the table.
(TIF)

S8 Fig Phenotyping iPSC-Vγ9Vδ2 T cells and donor blood-derived αβ T cells using flow cytometer. (TIF)

S9 Fig Cytotoxicity of iPSC-derived NK cells against zoledronic acid-treated cancer cells. NK cells were generated from a non-T cell-derived iPSC line (GDTA/NF-iPSC#3) and used for direct cytotoxicity against zoledronic acid-treated or non-treated colorectal adenocarcinoma line SW480 (mean ± SD, n = 3).
(TIF)

S1 Table Result summary of generation of iPSC lines from γδ T cells using three reprogramming strategies. (TIF)

S1 Video A 48-hour time-lapse video of coculture of γδ NKT and SW480 cancer cells. The video showed that γδ NKT cells eliminated most SW480 cells (a colorectal adenocarcinoma cell line) within first 12 hours.
(MP4)

S2 Video A 48-hour time-lapse video of coculture of γδ NKT and SW480 cancer cells at low cell density. The video clearly showed that the cancer cells were still actively proliferating if there were no attending γδ NKT cells (see the cancer cells in the 4^th^ yellow circle from the left on the top). Death of cancer cells only occurred when there were attending and contacting γδ NKT cells.
(MP4)

## References

[1] Lim WA, June CH. The Principles of Engineering Immune Cells to Treat Cancer. Cell. 2017;168(4):724–40. 10.1016/j.cell.2017.01.016 28187291 PMC5553442

[2] Roybal KT, Lim WA. Synthetic Immunology: Hacking Immune Cells to Expand Their Therapeutic Capabilities. Annual review of immunology. 2017;35:229–53. 10.1146/annurev-immunol-051116-052302 28446063 PMC5555230

[3] Themeli M, Kloss CC, Ciriello G, Fedorov VD, Perna F, Gonen M, et al Generation of tumor-targeted human T lymphocytes from induced pluripotent stem cells for cancer therapy. Nature biotechnology. 2013;31(10):928–33. 10.1038/nbt.2678 . 23934177 PMC5722218

[4] Magenau J, Runaas L, Reddy P. Advances in understanding the pathogenesis of graft-versus-host disease. British journal of haematology. 2016;173(2):190–205. 10.1111/bjh.13959 . 27019012

[5] Yu H, Sotillo E, Harrington C, Wertheim G, Paessler M, Maude SL, et al Repeated loss of target surface antigen after immunotherapy in primary mediastinal large B cell lymphoma. American journal of hematology. 2017;92(1):E11–E3. 10.1002/ajh.24594 . 27779774 PMC8620941

[6] Jacoby E, Nguyen SM, Fountaine TJ, Welp K, Gryder B, Qin H, et al CD19 CAR immune pressure induces B-precursor acute lymphoblastic leukaemia lineage switch exposing inherent leukaemic plasticity. Nature communications. 2016;7:12320 10.1038/ncomms12320 27460500 PMC4974466

[7] Bonneville M, Scotet E. Human Vgamma9Vdelta2 T cells: promising new leads for immunotherapy of infections and tumors. Current opinion in immunology. 2006;18(5):539–46. 10.1016/j.coi.2006.07.002 . 16870417

[8] Lamb LS Jr., Lopez RD. gammadelta T cells: a new frontier for immunotherapy? Biology of blood and marrow transplantation: journal of the American Society for Blood and Marrow Transplantation. 2005;11(3):161–8. 10.1016/j.bbmt.2004.11.015 . 15744234

[9] Deniger DC, Moyes JS, Cooper LJ. Clinical applications of gamma delta T cells with multivalent immunity. Frontiers in immunology. 2014;5:636 10.3389/fimmu.2014.00636 25566249 PMC4263175

[10] Rischer M, Pscherer S, Duwe S, Vormoor J, Jurgens H, Rossig C. Human gammadelta T cells as mediators of chimaeric-receptor redirected anti-tumour immunity. British journal of haematology. 2004;126(4):583–92. 10.1111/j.1365-2141.2004.05077.x . 15287953

[11] Morvan MG, Lanier LL. NK cells and cancer: you can teach innate cells new tricks. Nature reviews Cancer. 2016;16(1):7–19. 10.1038/nrc.2015.5 . 26694935

[12] Rezvani K, Rouce RH. The Application of Natural Killer Cell Immunotherapy for the Treatment of Cancer. Frontiers in immunology. 2015;6:578 10.3389/fimmu.2015.00578 26635792 PMC4648067

[13] Das H, Groh V, Kuijl C, Sugita M, Morita CT, Spies T, et al MICA engagement by human Vgamma2Vdelta2 T cells enhances their antigen-dependent effector function. Immunity. 2001;15(1):83–93. . 11485740 10.1016/s1074-7613(01)00168-6

[14] Rincon-Orozco B, Kunzmann V, Wrobel P, Kabelitz D, Steinle A, Herrmann T. Activation of V gamma 9V delta 2 T cells by NKG2D. Journal of immunology. 2005;175(4):2144–51. . 16081780 10.4049/jimmunol.175.4.2144

[15] Deng X, Terunuma H, Terunuma A, Takane T, Nieda M. Ex vivo-expanded natural killer cells kill cancer cells more effectively than ex vivo-expanded gammadelta T cells or alphabeta T cells. International immunopharmacology. 2014;22(2):486–91. 10.1016/j.intimp.2014.07.036 . 25131561

[16] Toutirais O, Cabillic F, Le Friec G, Salot S, Loyer P, Le Gallo M, et al DNAX accessory molecule-1 (CD226) promotes human hepatocellular carcinoma cell lysis by Vgamma9Vdelta2 T cells. European journal of immunology. 2009;39(5):1361–8. 10.1002/eji.200838409 . 19404979

[17] Pende D, Parolini S, Pessino A, Sivori S, Augugliaro R, Morelli L, et al Identification and molecular characterization of NKp30, a novel triggering receptor involved in natural cytotoxicity mediated by human natural killer cells. The Journal of experimental medicine. 1999;190(10):1505–16. 10562324 10.1084/jem.190.10.1505 PMC2195691

[18] Correia DV, Fogli M, Hudspeth K, da Silva MG, Mavilio D, Silva-Santos B. Differentiation of human peripheral blood Vdelta1+ T cells expressing the natural cytotoxicity receptor NKp30 for recognition of lymphoid leukemia cells. Blood. 2011;118(4):992–1001. 10.1182/blood-2011-02-339135 . 21633088

[19] Vitale M, Bottino C, Sivori S, Sanseverino L, Castriconi R, Marcenaro E, et al NKp44, a novel triggering surface molecule specifically expressed by activated natural killer cells, is involved in non-major histocompatibility complex-restricted tumor cell lysis. The Journal of experimental medicine. 1998;187(12):2065–72. 9625766 10.1084/jem.187.12.2065 PMC2212362

[20] von Lilienfeld-Toal M, Nattermann J, Feldmann G, Sievers E, Frank S, Strehl J, et al Activated gammadelta T cells express the natural cytotoxicity receptor natural killer p 44 and show cytotoxic activity against myeloma cells. Clinical and experimental immunology. 2006;144(3):528–33. 10.1111/j.1365-2249.2006.03078.x 16734623 PMC1941970

[21] Van Acker HH, Anguille S, Willemen Y, Van den Bergh JM, Berneman ZN, Lion E, et al Interleukin-15 enhances the proliferation, stimulatory phenotype, and antitumor effector functions of human gamma delta T cells. Journal of hematology & oncology. 2016;9(1):101 10.1186/s13045-016-0329-3 27686372 PMC5041439

[22] Kreslavsky T, von Boehmer H. gammadeltaTCR ligands and lineage commitment. Seminars in immunology. 2010;22(4):214–21. 10.1016/j.smim.2010.04.001 20447836 PMC2912151

[23] Nishimura T, Kaneko S, Kawana-Tachikawa A, Tajima Y, Goto H, Zhu D, et al Generation of rejuvenated antigen-specific T cells by reprogramming to pluripotency and redifferentiation. Cell stem cell. 2013;12(1):114–26. 10.1016/j.stem.2012.11.002 . 23290140

[24] Vizcardo R, Masuda K, Yamada D, Ikawa T, Shimizu K, Fujii S, et al Regeneration of human tumor antigen-specific T cells from iPSCs derived from mature CD8(+) T cells. Cell stem cell. 2013;12(1):31–6. 10.1016/j.stem.2012.12.006 . 23290135

[25] Zeng J, Tang SY, Toh LL, Wang S. Generation of "Off-the-Shelf" Natural Killer Cells from Peripheral Blood Cell-Derived Induced Pluripotent Stem Cells. Stem cell reports. 2017;9(6):1796–812. 10.1016/j.stemcr.2017.10.020 29173894 PMC5785702

[26] Zeng J, Shahbazi M, Wu C, Toh HC, Wang S. Enhancing immunostimulatory function of human embryonic stem cell-derived dendritic cells by CD1d overexpression. Journal of immunology. 2012;188(9):4297–304. 10.4049/jimmunol.1102343 . 22407918

[27] Zeng J, Wang S. Human dendritic cells derived from embryonic stem cells stably modified with CD1d efficiently stimulate antitumor invariant natural killer T cell response. Stem cells translational medicine. 2014;3(1):69–80. 10.5966/sctm.2013-0070 24292792 PMC3902285

[28] Zeng J, Wu C, Wang S. Antigenically Modified Human Pluripotent Stem Cells Generate Antigen-Presenting Dendritic Cells. Scientific reports. 2015;5:15262 10.1038/srep15262 26471005 PMC4608011

[29] Eyquem J, Mansilla-Soto J, Giavridis T, van der Stegen SJ, Hamieh M, Cunanan KM, et al Targeting a CAR to the TRAC locus with CRISPR/Cas9 enhances tumour rejection. Nature. 2017;543(7643):113–7. 10.1038/nature21405 28225754 PMC5558614

[30] Watanabe D, Koyanagi-Aoi M, Taniguchi-Ikeda M, Yoshida Y, Azuma T, Aoi T. The Generation of Human gammadeltaT Cell-Derived Induced Pluripotent Stem Cells from Whole Peripheral Blood Mononuclear Cell Culture. Stem cells translational medicine. 2018;7(1):34–44. 10.1002/sctm.17-0021 29164800 PMC5746152

[31] Ali SA, Shi V, Maric I, Wang M, Stroncek DF, Rose JJ, et al T cells expressing an anti-B-cell maturation antigen chimeric antigen receptor cause remissions of multiple myeloma. Blood. 2016;128(13):1688–700. 10.1182/blood-2016-04-711903 27412889 PMC5043125

[32] Marcus A, Gowen BG, Thompson TW, Iannello A, Ardolino M, Deng W, et al Recognition of tumors by the innate immune system and natural killer cells. Advances in immunology. 2014;122:91–128. 10.1016/B978-0-12-800267-4.00003-1 24507156 PMC4228931

[33] Pazina T, Shemesh A, Brusilovsky M, Porgador A, Campbell KS. Regulation of the Functions of Natural Cytotoxicity Receptors by Interactions with Diverse Ligands and Alterations in Splice Variant Expression. Frontiers in immunology. 2017;8:369 10.3389/fimmu.2017.00369 28424697 PMC5371597

[34] Gober HJ, Kistowska M, Angman L, Jeno P, Mori L, De Libero G. Human T cell receptor gammadelta cells recognize endogenous mevalonate metabolites in tumor cells. The Journal of experimental medicine. 2003;197(2):163–8. 10.1084/jem.20021500 12538656 PMC2193814

[35] Raulet DH, Marcus A, Coscoy L. Dysregulated cellular functions and cell stress pathways provide critical cues for activating and targeting natural killer cells to transformed and infected cells. Immunological reviews. 2017;280(1):93–101. 10.1111/imr.12600 29027233 PMC5687887

[36] Ren J, Liu X, Fang C, Jiang S, June CH, Zhao Y. Multiplex Genome Editing to Generate Universal CAR T Cells Resistant to PD1 Inhibition. Clinical cancer research: an official journal of the American Association for Cancer Research. 2017;23(9):2255–66. 10.1158/1078-0432.CCR-16-1300 27815355 PMC5413401

[37] Ren J, Zhang X, Liu X, Fang C, Jiang S, June CH, et al A versatile system for rapid multiplex genome-edited CAR T cell generation. Oncotarget. 2017;8(10):17002–11. 10.18632/oncotarget.15218 28199983 PMC5370017

[38] Maude SL, Frey N, Shaw PA, Aplenc R, Barrett DM, Bunin NJ, et al Chimeric antigen receptor T cells for sustained remissions in leukemia. The New England journal of medicine. 2014;371(16):1507–17. 10.1056/NEJMoa1407222 25317870 PMC4267531

[39] Grupp SA, Kalos M, Barrett D, Aplenc R, Porter DL, Rheingold SR, et al Chimeric antigen receptor-modified T cells for acute lymphoid leukemia. The New England journal of medicine. 2013;368(16):1509–18. 10.1056/NEJMoa1215134 23527958 PMC4058440

[40] Kalos M, Levine BL, Porter DL, Katz S, Grupp SA, Bagg A, et al T cells with chimeric antigen receptors have potent antitumor effects and can establish memory in patients with advanced leukemia. Science translational medicine. 2011;3(95):95ra73 10.1126/scitranslmed.3002842 21832238 PMC3393096

