# Supplementary figures and images for: Derivation of mimetic γδ T cells endowed with cancer recognition receptors from reprogrammed γδ T cell
Source: PLoS One. 2019 May 9;14(5):e0216815. doi: 10.1371/journal.pone.0216815 (PMC6508724; doi:10.1371/journal.pone.0216815)

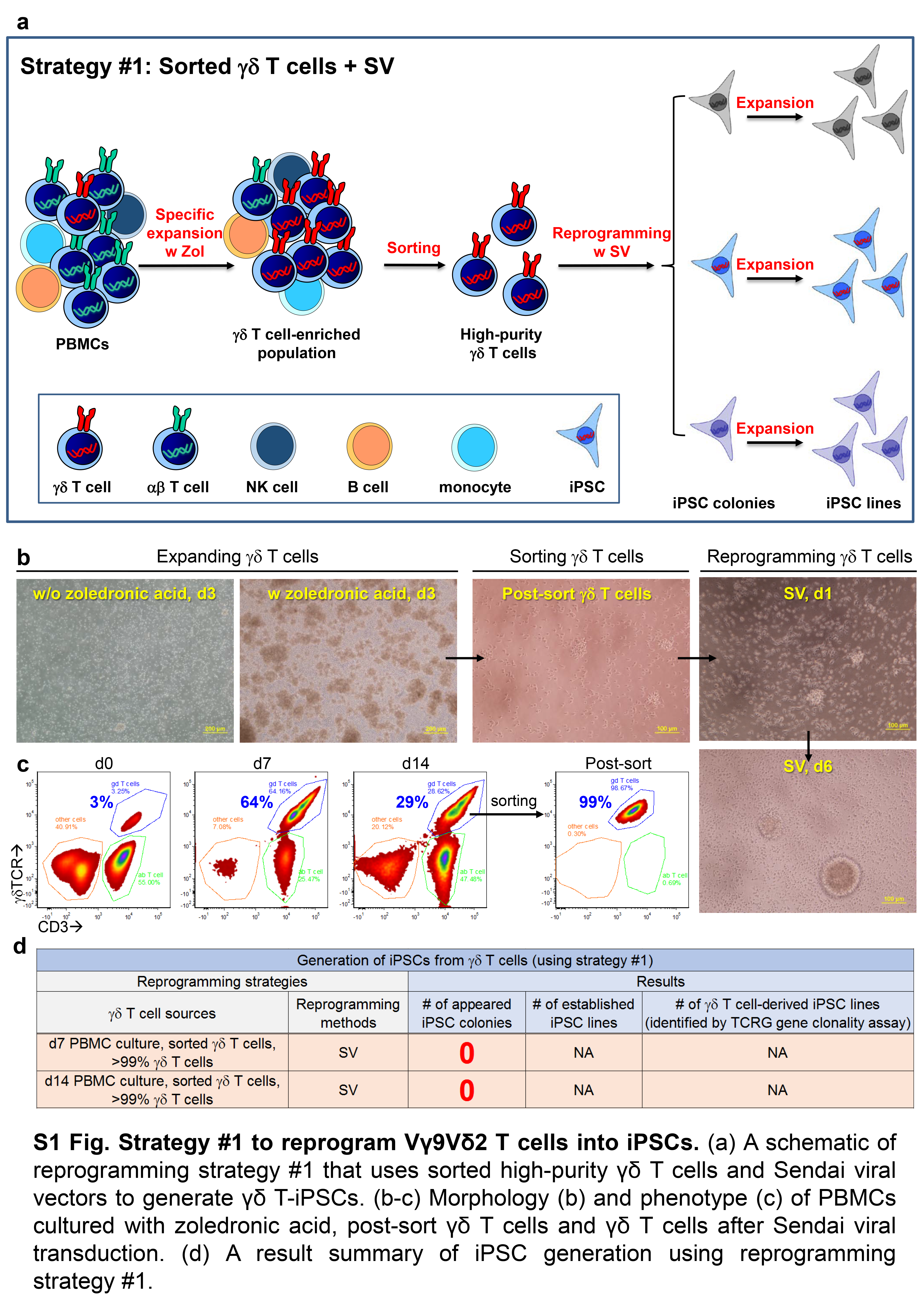

Supplement: S1 Fig — (a) A schematic of reprogramming strategy #1 that uses sorted high-purity γδ T cells and Sendai viral vectors to generate γδ T-iPSCs. (b-c) Morphology (b) and phenotype (c) of PBMCs cultured with zoledronic acid, post-sort γδ T cells and γδ T cells after Sendai viral transduction. (d) A result summary of iPSC generation using reprogramming strategy #1. (TIF) [file pone.0216815.s001.tif]

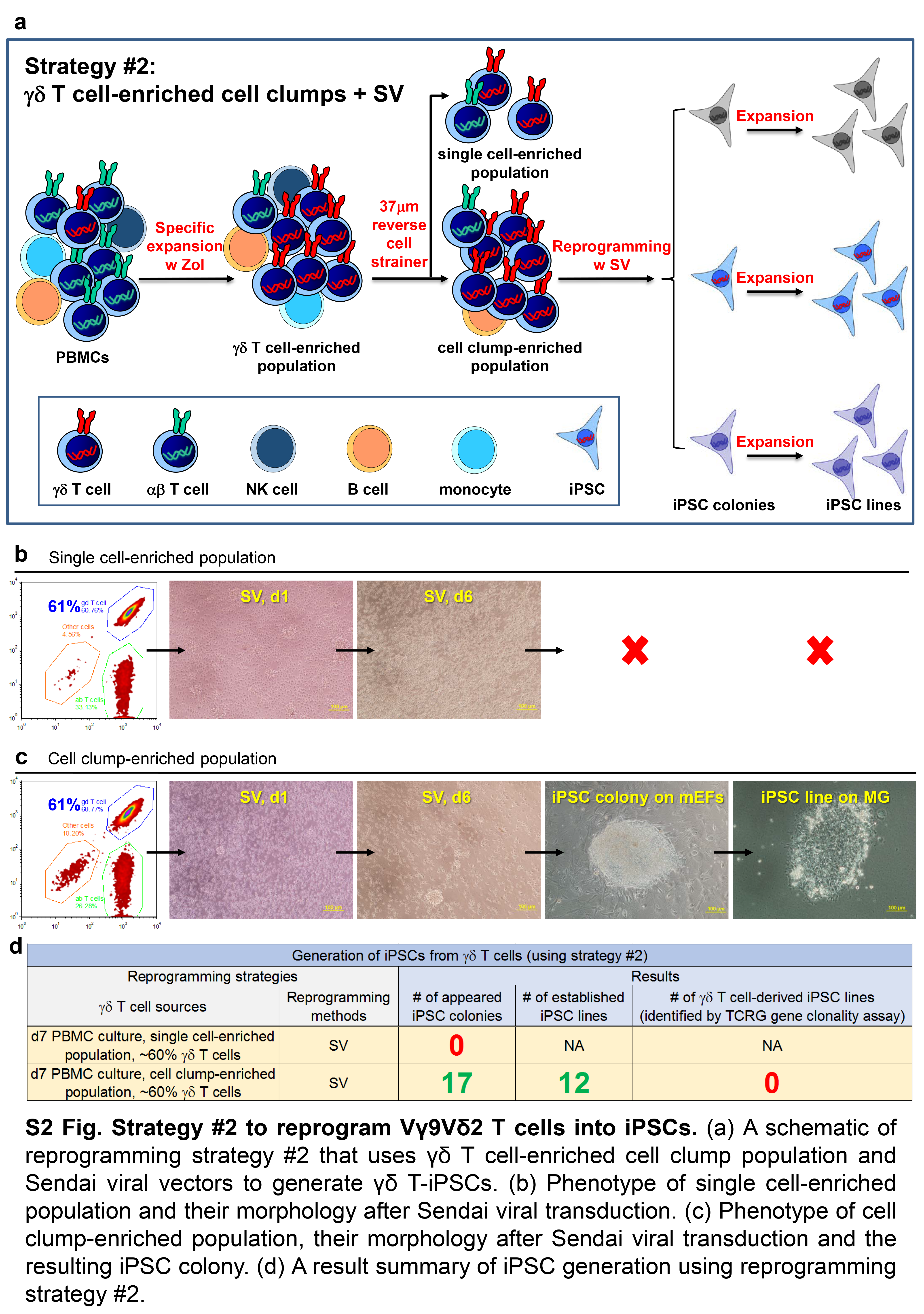

Supplement: S2 Fig — (a) A schematic of reprogramming strategy #2 that uses γδ T cell-enriched cell clump population and Sendai viral vectors to generate γδ T-iPSCs. (b) Phenotype of single cell-enriched population and their morphology after Sendai viral transduction. (c) Phenotype of cell clump-enriched population, their morphology after Sendai viral transduction and the resulting iPSC colony. (d) A result summary of iPSC generation using reprogramming strategy #2. (TIF) [file pone.0216815.s002.tif]

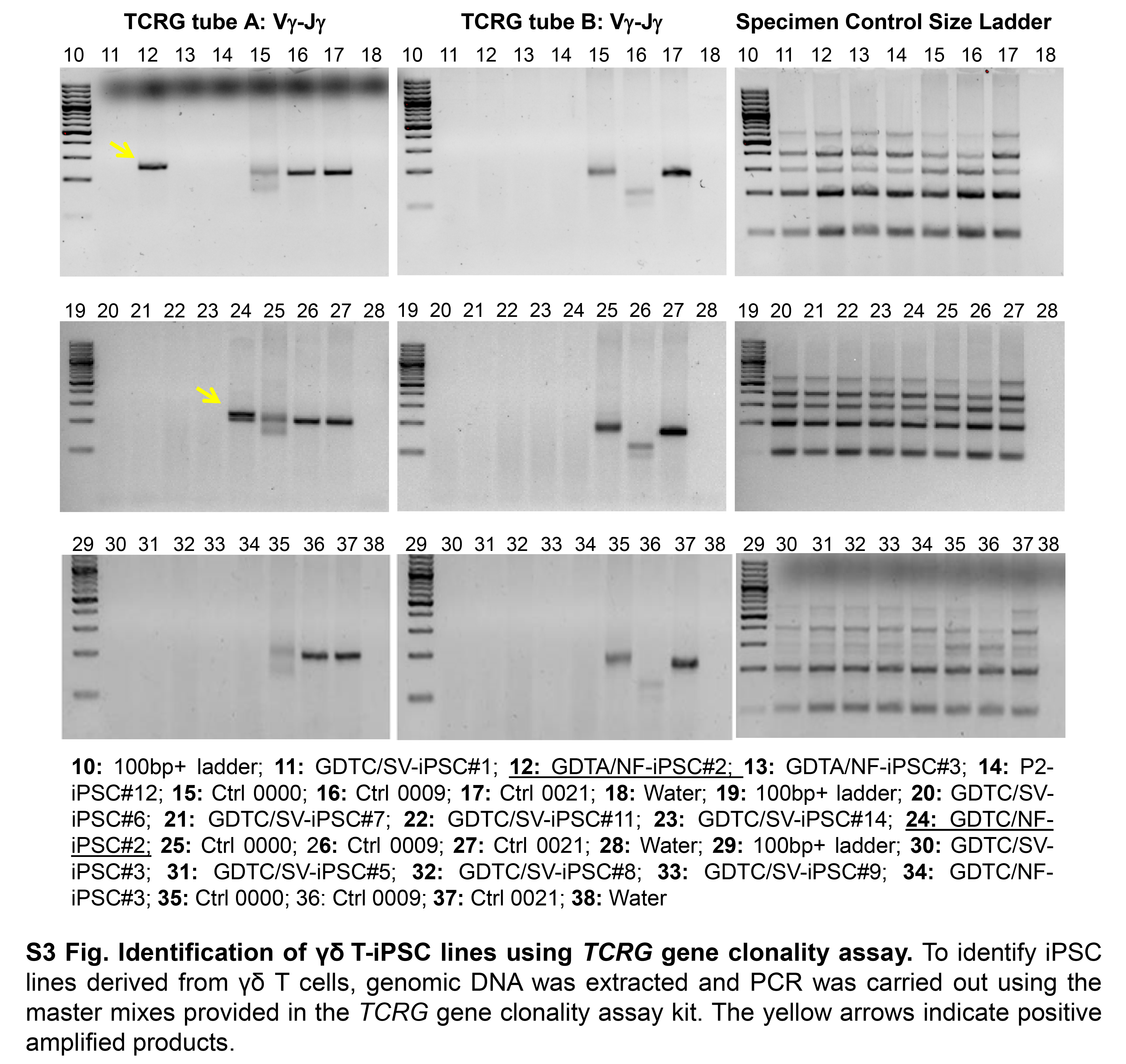

Supplement: S3 Fig — To identify iPSC lines derived from γδ T cells, genomic DNA was extracted and PCR was carried out using the master mixes provided in the TCRG gene clonality assay kit. The yellow arrows indicate positive amplified products. (TIF) [file pone.0216815.s003.tif]

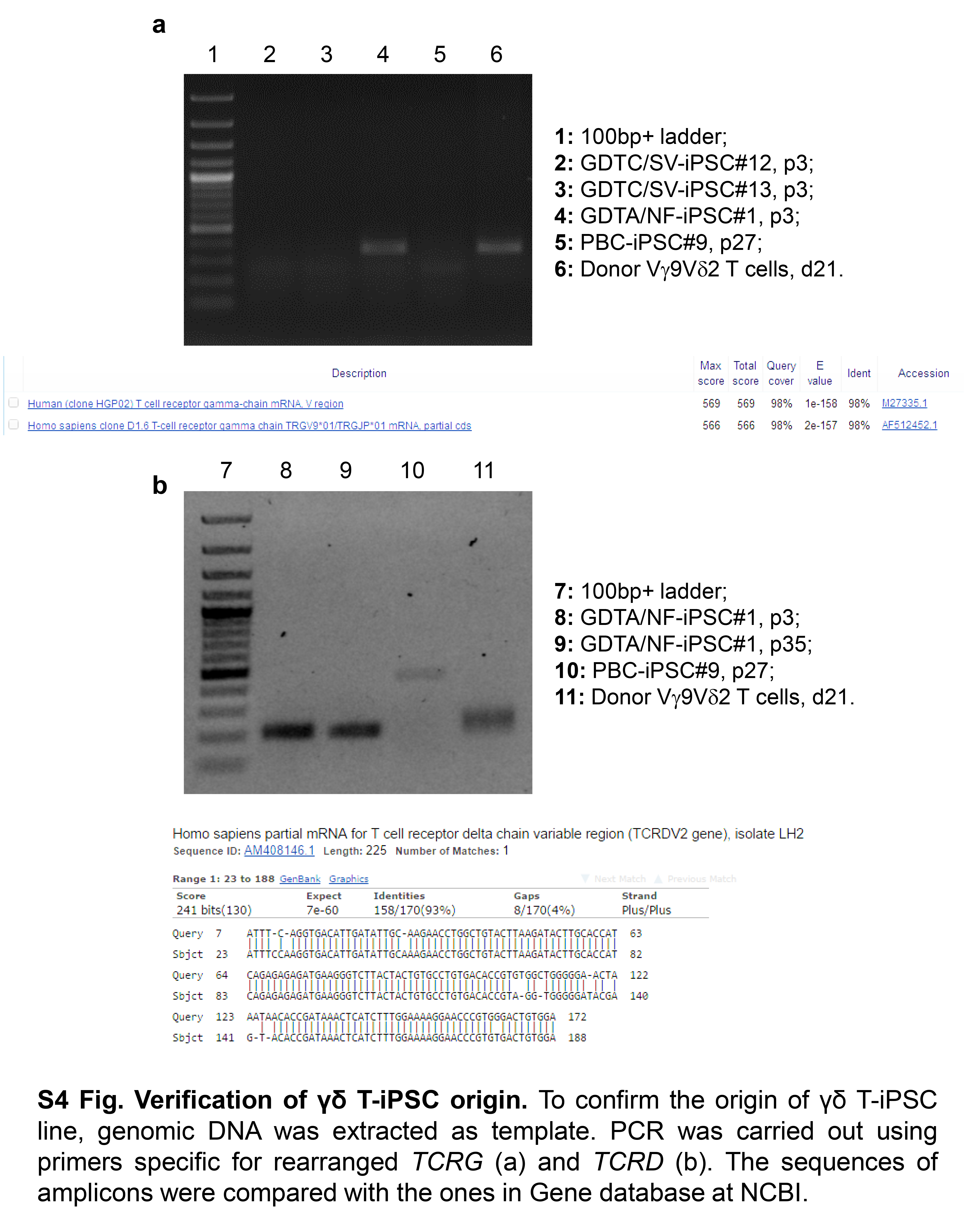

Supplement: S4 Fig — To confirm the origin of γδ T-iPSC line, genomic DNA was extracted as template. PCR was carried out using primers specific for rearranged TCRG (a) and TCRD (b). The sequences of amplicons were compared with the ones in Gene database at NCBI. (TIF) [file pone.0216815.s004.tif]

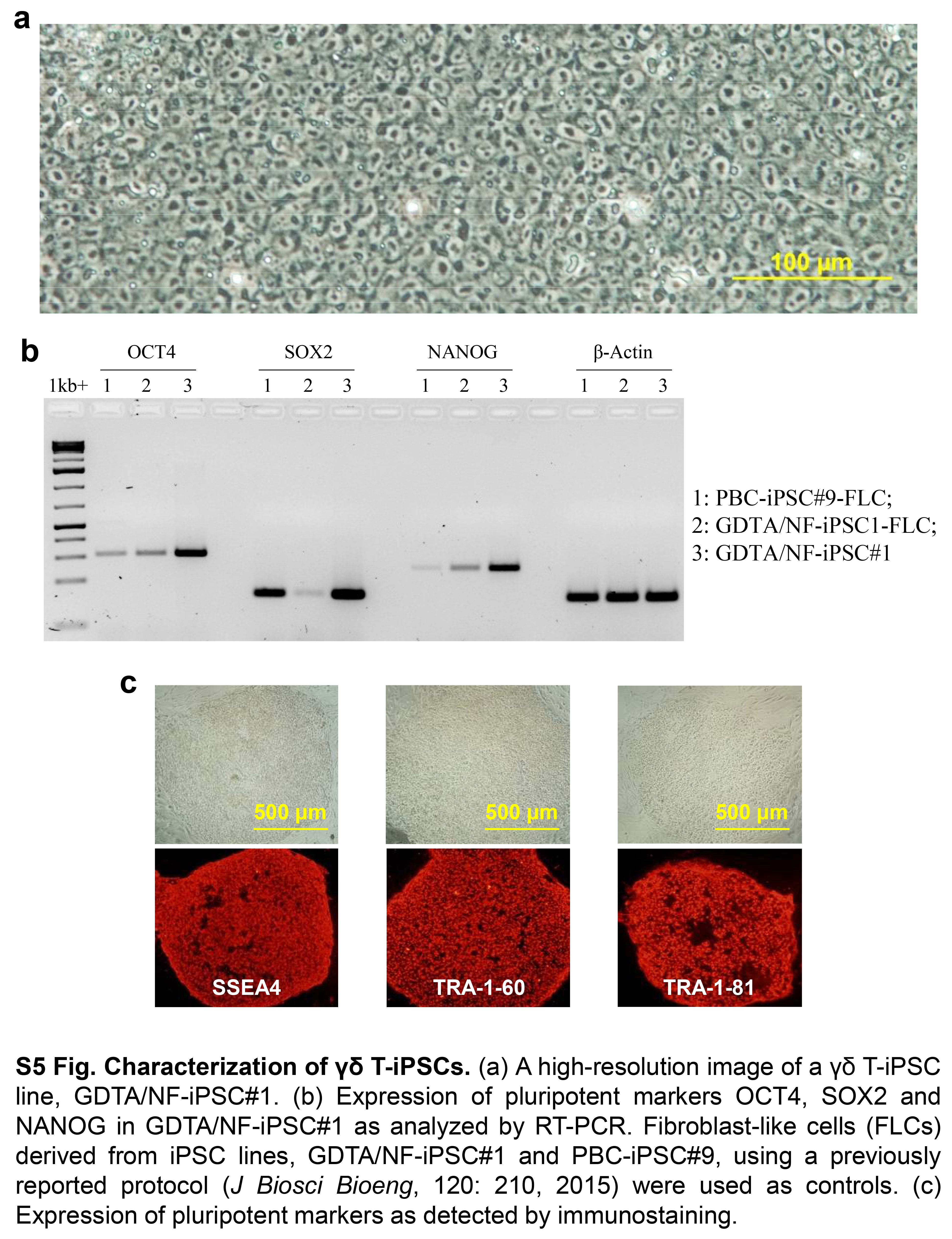

Supplement: S5 Fig — (a) A high resolution image of a γδ T-iPSC line, GDTA/NF-iPSC#1. (b) Expression of pluripotent markers OCT4, SOX2 and NANOG in GDTA/NF-iPSC#1 as analyzed by RT-PCR. Fibroblast-like cells (FLCs) derived from iPSC lines, GDTA/NF-iPSC#1 and PBC-iPSC#9, using a previously reported protocol (J Biosci Bioeng, 120: 210, 2015) were used as controls. (c) Expression of pluripotent markers as detected by immunostaining. (TIF) [file pone.0216815.s005.tif]

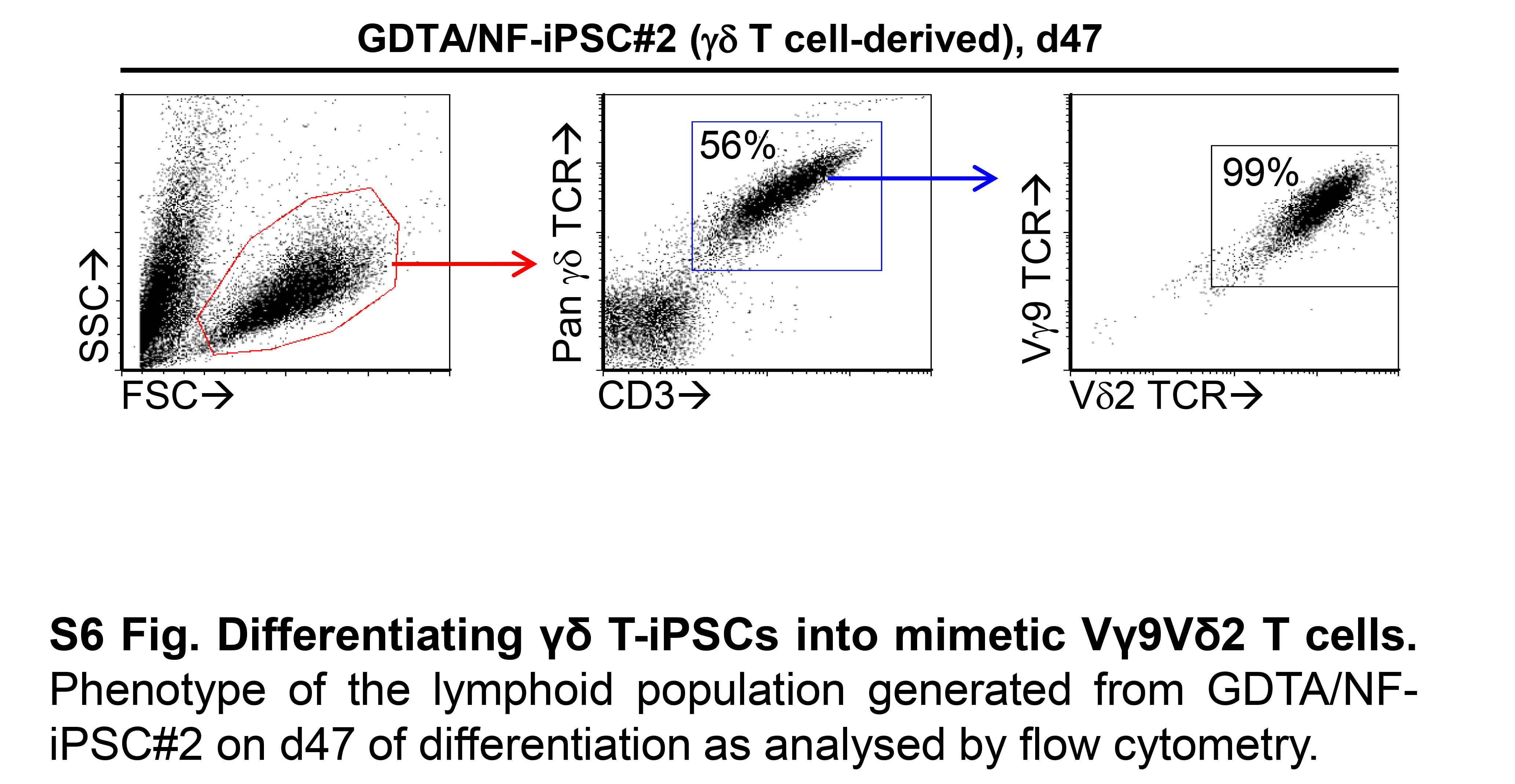

Supplement: S6 Fig — Phenotype of the lymphoid population generated from GDTA/NF-iPSC#2 on d47 of differentiation as analysed by flow cytometry. (TIF) [file pone.0216815.s006.tif]

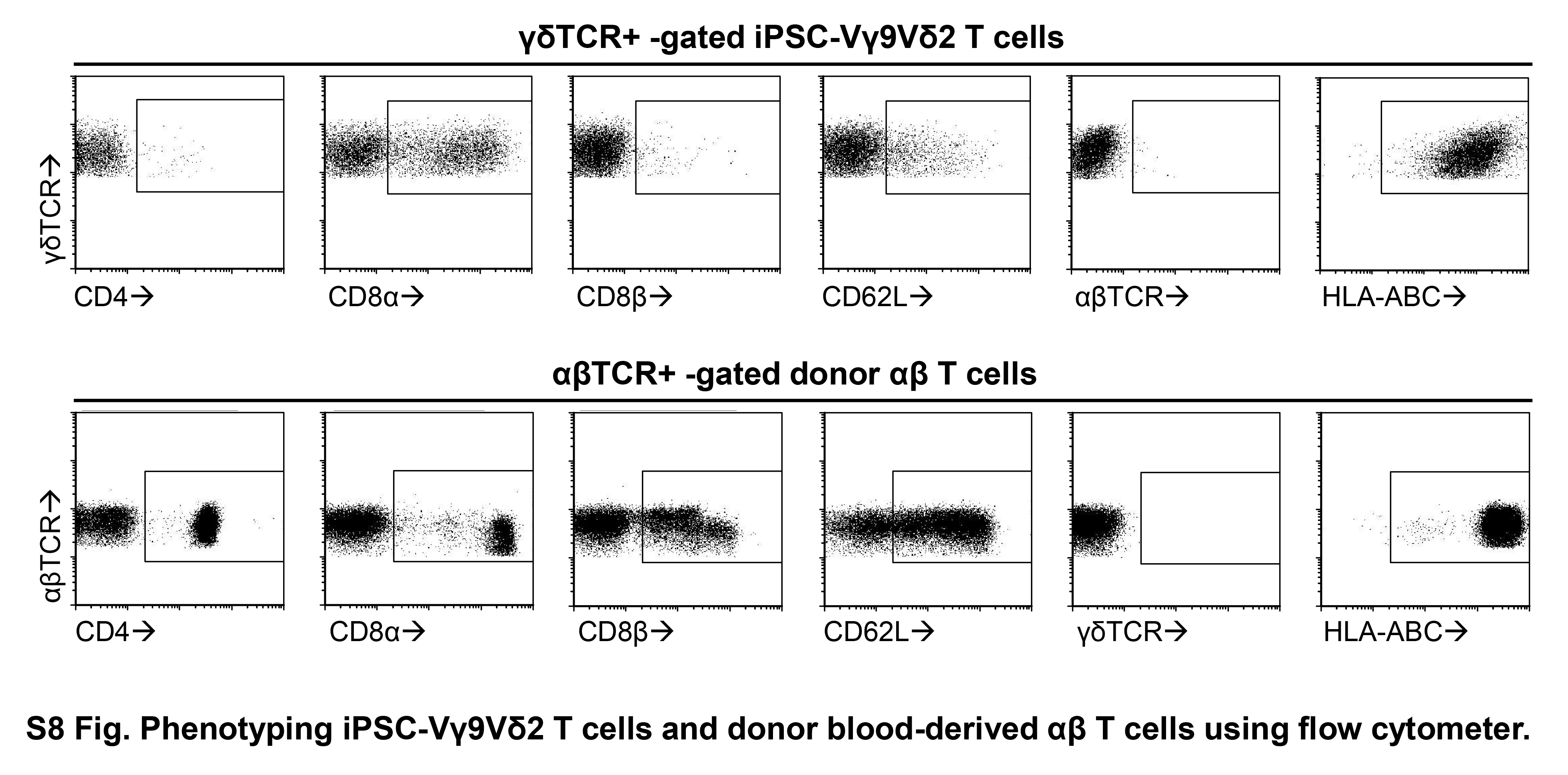

Supplement: S8 Fig — (TIF) [file pone.0216815.s008.tif]

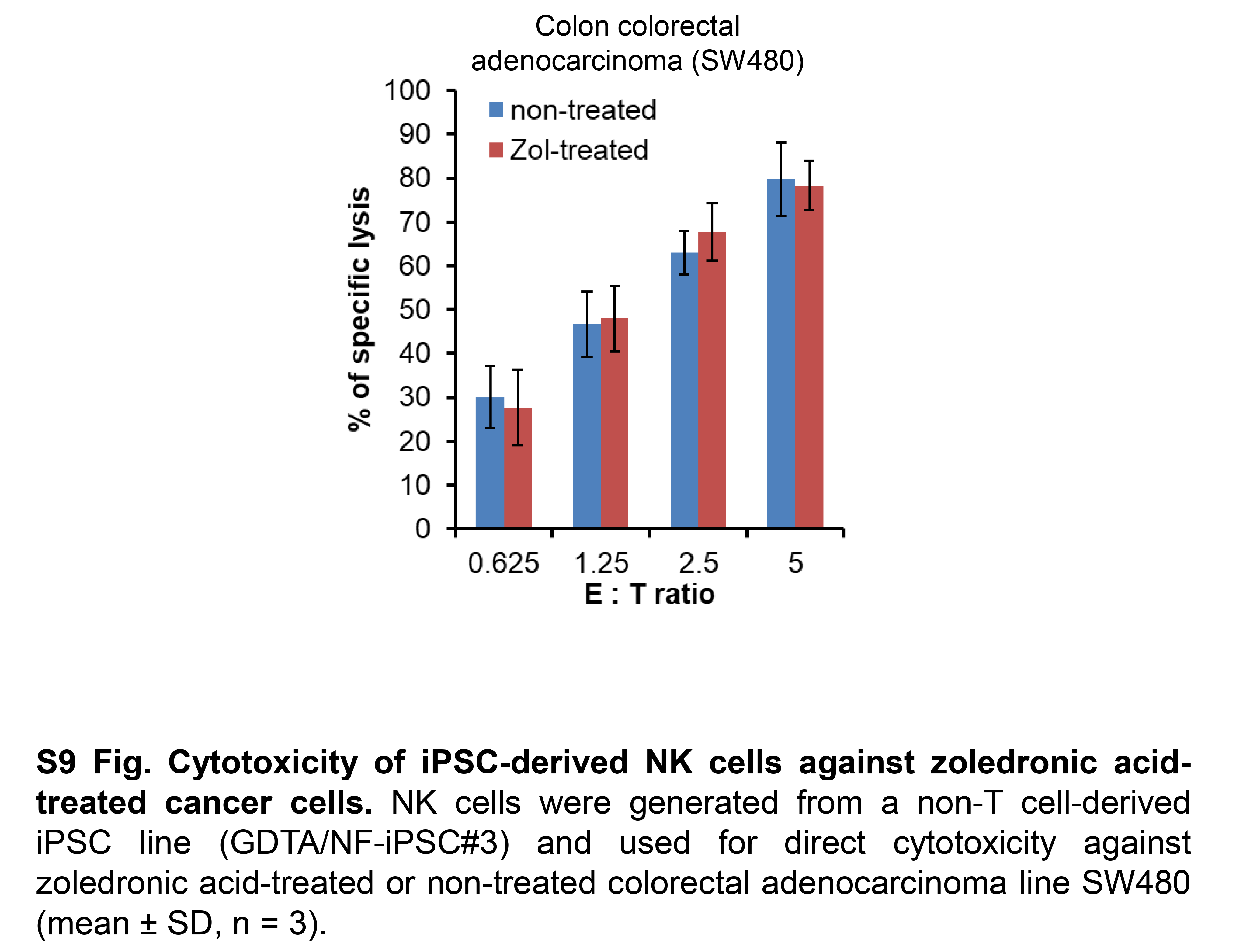

Supplement: S9 Fig — NK cells were generated from a non-T cell-derived iPSC line (GDTA/NF-iPSC#3) and used for direct cytotoxicity against zoledronic acid-treated or non-treated colorectal adenocarcinoma line SW480 (mean ± SD, n = 3). (TIF) [file pone.0216815.s009.tif]

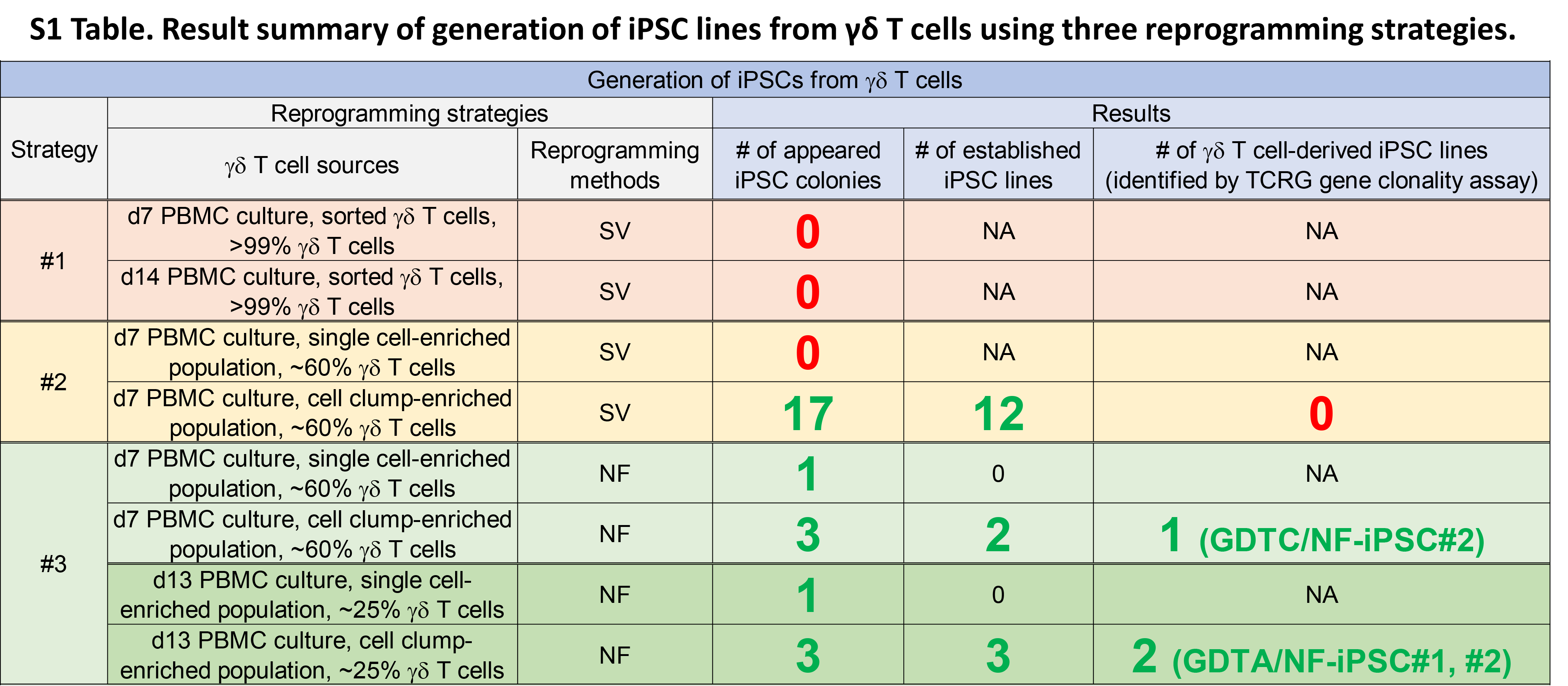

Supplement: S1 Table — (TIF) [file pone.0216815.s010.tif]
